# SND1 binds SARS-CoV-2 negative-sense RNA and promotes viral RNA synthesis through NSP9

**DOI:** 10.1016/j.cell.2023.09.002

**Published:** 2023-10-26

**Authors:** Nora Schmidt, Sabina Ganskih, Yuanjie Wei, Alexander Gabel, Sebastian Zielinski, Hasmik Keshishian, Caleb A. Lareau, Liv Zimmermann, Jana Makroczyova, Cadence Pearce, Karsten Krey, Thomas Hennig, Sebastian Stegmaier, Lambert Moyon, Marc Horlacher, Simone Werner, Jens Aydin, Marco Olguin-Nava, Ramya Potabattula, Anuja Kibe, Lars Dölken, Redmond P. Smyth, Neva Caliskan, Annalisa Marsico, Christine Krempl, Jochen Bodem, Andreas Pichlmair, Steven A. Carr, Petr Chlanda, Florian Erhard, Mathias Munschauer

**Affiliations:** 1 Helmholtz Institute for RNA-based Infection Research (HIRI), Helmholtz Centre for Infection Research (HZI), Würzburg, Germany; 2 Broad Institute of MIT and Harvard, Cambridge, MA, USA; 3 Program in Computational and System Biology, Memorial Sloan Kettering Cancer Center, New York, NY, USA; 4 Schaller Research Group, Department of Infectious Diseases, Virology, Heidelberg University Hospital, Heidelberg, Germany; 5 School of Medicine, Institute of Virology, Technical University of Munich, Munich, Germany; 6 Institute for Virology and Immunobiology, Julius-Maximilians-University Würzburg, Würzburg, Germany; 7 Computational Health Center, Helmholtz Center Munich, Munich, Germany; 8 Institute of Human Genetics, Julius-Maximilians-University Würzburg, Würzburg, Germany; 9 German Center for Infection Research (DZIF), Munich Partner Site, Munich, Germany; 10 Faculty for Computer and Data Science, University of Regensburg, Regensburg, Germany; 11 Faculty of Medicine, Julius-Maximilians-University Würzburg, Würzburg, Germany

**Keywords:** RNA binding proteins, SARS-CoV-2, virus host interactions, proteomics, RNA virus, systems biology, RNA biology, RNA interactome, host factors, omics technologies

## Abstract

Regulation of viral RNA biogenesis is fundamental to productive SARS-CoV-2 infection. To characterize host RNA-binding proteins (RBPs) involved in this process, we biochemically identified proteins bound to genomic and subgenomic SARS-CoV-2 RNAs. We find that the host protein SND1 binds the 5′ end of negative-sense viral RNA and is required for SARS-CoV-2 RNA synthesis. SND1-depleted cells form smaller replication organelles and display diminished virus growth kinetics. We discover that NSP9, a viral RBP and direct SND1 interaction partner, is covalently linked to the 5′ ends of positive- and negative-sense RNAs produced during infection. These linkages occur at replication-transcription initiation sites, consistent with NSP9 priming viral RNA synthesis. Mechanistically, SND1 remodels NSP9 occupancy and alters the covalent linkage of NSP9 to initiating nucleotides in viral RNA. Our findings implicate NSP9 in the initiation of SARS-CoV-2 RNA synthesis and unravel an unsuspected role of a cellular protein in orchestrating viral RNA production.

## Highlights

• SND1 is required for nascent SARS-CoV-2 RNA synthesis early during infection
• SND1 directly interacts with NSP9 and both proteins bind negative-sense viral RNA
• NSP9 is covalently linked to viral RNA at initiation sites, indicating protein priming
• SND1 modulates the covalent linkage of NSP9 to positive- and negative-sense viral RNA

## Graphical abstract

**Figure:**
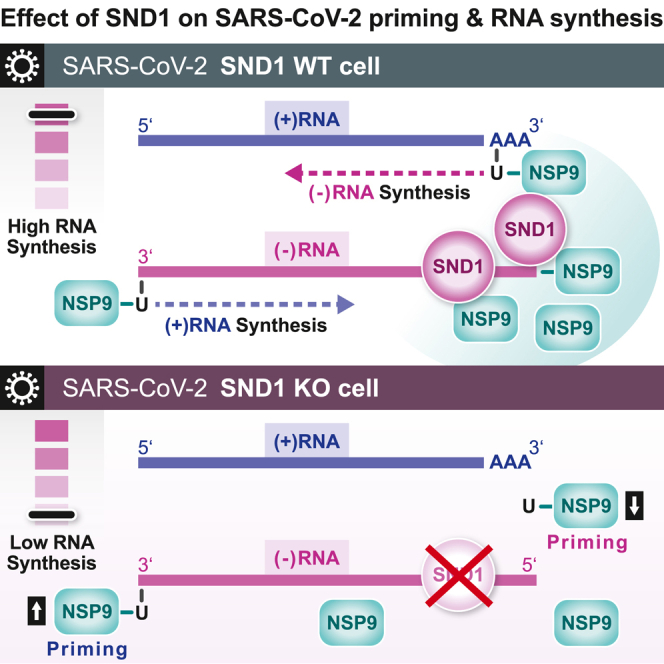

## Teaser

Mapping of subgenome-resolved SARS-CoV-2 RNA-protein interactions reveals that the host protein SND1 binds negative-sense SARS-CoV-2 RNA and promotes viral RNA synthesis by recruiting NSP9, which likely serves as a protein primer for RNA production.

## Introduction

SARS-CoV-2 is an enveloped, positive-sense, single-stranded RNA virus that, upon infection of a cell, deploys a 5′ capped and 3′-polyadenylated RNA genome to directly engage the host protein synthesis machinery.^1^ During the initial stage of genomic RNA (gRNA) translation, host ribosomes together with translation factors and RNA-binding proteins (RBPs) produce two polyproteins encoded by ORF1a and ORF1ab.^2^ ORF1ab contains 16 non-structural proteins (NSPs) that are proteolytically processed to produce enzymes required for viral RNA synthesis.^3^ While NSP2 to NSP11 facilitate formation of the viral replication-transcription complex (RTC), NSP12 to NSP16 provide core enzymatic activities for viral RNA synthesis, RNA proofreading, and RNA modification.^1^^,^^3^ Coronavirus RTCs are associated with virus-induced double-membrane vesicles (DMVs) and viral RNA synthesis occurs within or near these membrane structures. In addition to NSPs, SARS-CoV-2 encodes four structural proteins (nucleocapsid [N], envelope [E], membrane [M], and spike [S]) and eight accessory proteins (ORF3a–ORF9b), expressed from a nested set of 5′ and 3′ co-terminal subgenomic mRNAs (sgmRNAs).

The biogenesis of sgmRNAs starts at the 3′ end of the viral genome and involves a discontinuous transcription mechanism that produces negative-sense RNA intermediates from the gRNA template.^4^ During discontinuous transcription, a copy of the genomic 5′ leader is fused to all subgenomic RNAs by a template-switching mechanism.^2^ Negative-sense subgenomic RNAs subsequently become templates for transcribing positive-sense sgmRNAs. Full-length copies of the viral genome are produced from negative-sense RNA templates generated by continuous RNA synthesis. The mechanism of subgenomic RNA transcription is controlled by *cis*-regulatory sequence elements, such as transcription regulatory sequences (TRSs), that engage in RNA-RNA interactions and work together with RBPs of virus and host to facilitate viral gene expression.^2^^,^^4^^,^^5^

While the core viral proteins required to synthesize viral RNA are known, we lack an understanding of the involvement of host proteins and their impact on viral RNA biogenesis. Remarkably, the activity of isolated coronavirus RTCs is dependent on uncharacterized cytoplasmic host factor(s) *in vitro*.^6^ Factors capable of stimulating viral RNA biogenesis are likely proteins that bind viral RNA. While several recent studies have identified cellular factors associated with all SARS-CoV-2 RNAs,^7^^,^^8^^,^^9^^,^^10^ these studies do not reveal proteins that specifically bind certain viral RNA subtypes. Since SARS-CoV-2 genomic and subgenomic RNAs differ in their biogenesis mechanism and molecular function, they likely have distinct host factor dependencies and interaction profiles. To characterize the molecular interactions of different SARS-CoV-2 RNA subtypes, we used a multistep RNA antisense purification (RAP) strategy to resolve interactomes of genomic and subgenomic SARS-CoV-2 RNAs. Focusing on factors with a binding preference for subgenomic RNAs, we find that the host protein staphylococcal nuclease domain-containing protein 1 (SND1) recognizes negative-sense RNA of SARS-CoV-2 and is required for viral RNA synthesis in human cells. The function of SND1 in SARS-CoV-2 replication depends on its interaction with NSP9 and loss of SND1 leads to imbalanced NSP9 occupancy at replication-transcription initiation sites, where NSP9 is covalently linked to the 5′ ends of positive and negative-sense viral RNAs. These findings implicate NSP9 in priming of viral RNA synthesis and reveal an unexpected role of SND1 in viral RNA biogenesis.

## Results

### Biochemical separation of SARS-CoV-2 genomic and subgenomic RNA

**Figure 1:**
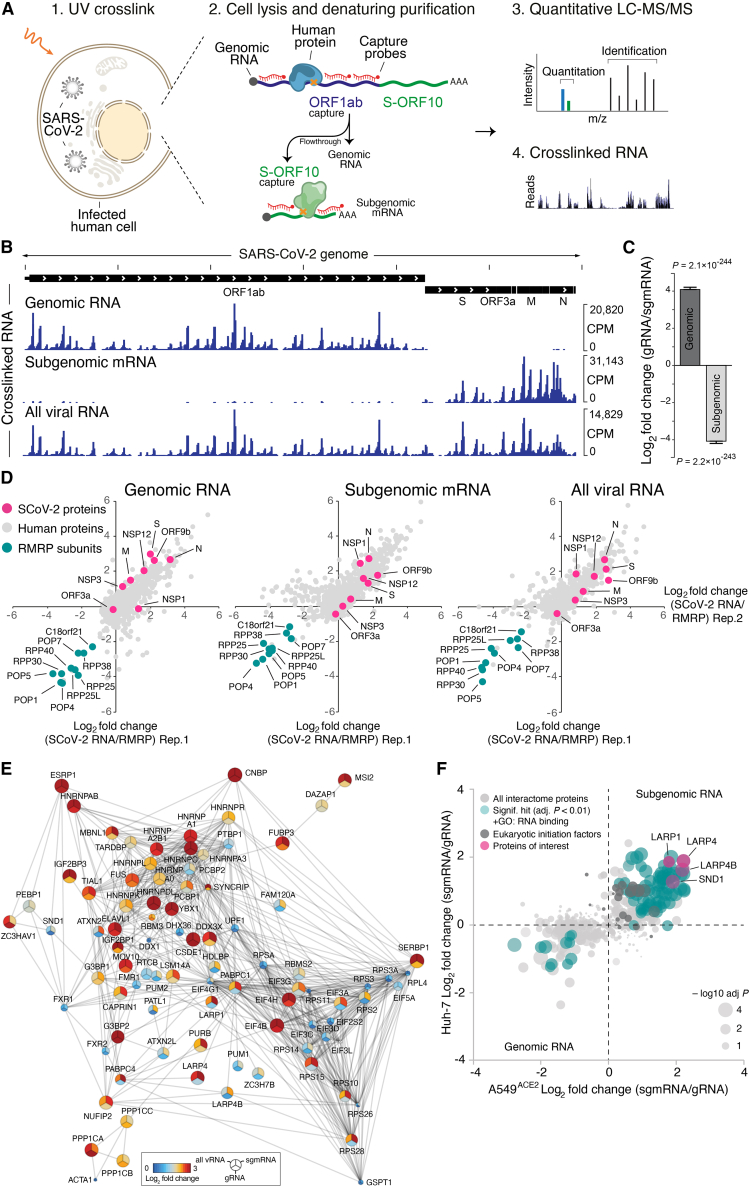
The SARS-CoV-2 RNA-protein interactome at subgenome resolution (A) Outline of RAP-MS workflow to identify proteins bound to SARS-CoV-2 genomic RNA (gRNA) and subgenomic mRNA (sgmRNA). (B) Sequencing of RNA crosslinked to proteins purified with RAP-MS. (C) Enrichment of reads mapping to SARS-CoV-2 genomic and subgenomic RNA in gRNA relative to sgmRNA purifications ± SE. p values determined by Wald test. (D) Quantification of SARS-CoV-2 RNA interacting proteins relative to RMRP interacting proteins in antisense purifications of indicated RNA species. Log_2_ fold changes from two biological replicates in Huh-7 cells are shown. Gray, all detected proteins; magenta, SARS-CoV-2 proteins; teal, RMRP components. (E) Protein-protein association network for consensus SARS-CoV-2 RNA interactome based on interactions in STRING v11.^13^ Coloring indicates RAP-MS enrichment in Huh-7 cells. Nodes are scaled to significance. (F) Quantification of sgmRNA relative to gRNA interactomes. Average log_2_ fold changes in A549^ACE2^ cells (x axis) and Huh-7 cells (y axis) are shown. Circles are scaled to significance. Keratins and carboxylases were removed. (E and F) Combined p values calculated using Fisher’s method for combined probability and adjusted using Benjamini-Hochberg procedure. See also Figure S1 and Tables S1 and S2.

To achieve a clean biochemical separation of SARS-CoV-2 gRNA and sgmRNA, we modified the RNA capture strategy implemented in RAP and mass spectrometry (RAP-MS)^8^^,^^11^^,^^12^ by constructing antisense capture probes that hybridize to the ORF1ab region, which is not present in sgmRNAs (Figure 1A). This initial capture step enriches gRNAs, while sgmRNAs remain in the flowthrough. After removal of excess capture probes, we add a different pool of antisense probes that hybridize to the S-ORF10 region and capture all SARS-CoV-2 sgmRNAs (Figure 1A).

**Figure S1:**
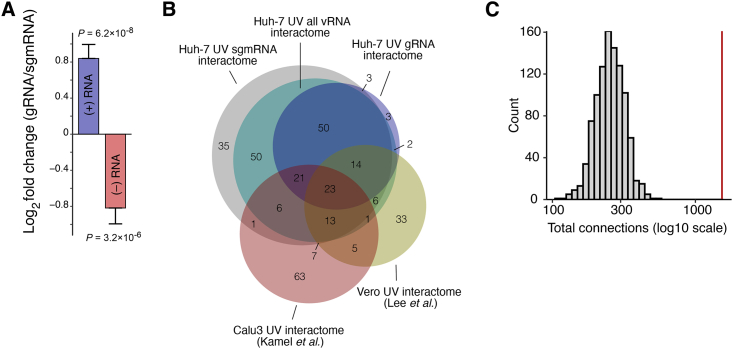
Analysis of subgenome-resolved SARS-CoV-2 RNA-protein interactions, related to Figure 1 (A) Enrichment of reads mapping to SARS-CoV-2 positive-sense or negative-sense RNA in gRNA purifications relative to sgmRNA purifications ± SE. p values determined by Wald test. (B) Venn diagram comparing two UV-based SARS-CoV-2 RNA interactomes^7^^,^^9^ to the subgenome-resolved RNA interactome presented in this study (Huh-7 cells, FDR < 5%, Table S2). Only human proteins are shown. (C) Total number of connections observed in protein-protein association network constructed based on consensus SARS-CoV-2 RNA interactome (red line, 1,280 connections), compared to number of connections observed in random networks of equal size (gray bars, mean 259.9 connections) using random sampling of proteins detected in proteome measurements (1,000 permutations).

Using this strategy, we performed RAP-MS in Huh-7 cells at 24 h post-infection (hpi).^8^ We first extracted UV-crosslinked RNA from proteins purified by RAP-MS and sequenced the recovered RNA.^8^ When purifying gRNAs and sgmRNAs separately, crosslinked RNA was overwhelmingly recovered from targeted RNA regions, confirming the successful separation of genomic from subgenomic RNA (Figures 1B and 1C). We also detected reads derived from negative-sense RNA, which were enriched in sgmRNA relative to gRNA purifications (Figure S1A). Hence, sgmRNA purifications contain proteins crosslinked to positive and negative-sense RNA, a binding pattern expected for factors contacting both RNA strands, such as RTC components or proteins involved in viral RNA biogenesis. We next subjected proteins recovered from each RNA purification to tandem mass tag (TMT)-labeling and quantitative mass spectrometry. As described previously,^8^ we quantitatively compared the protein content of SARS-CoV-2 RNA purifications to purifications of the RNA component of mitochondrial RNA processing endoribonuclease (RMRP)^12^^,^^14^ (Figure 1D). This strategy yields specific SARS-CoV-2 RNA binders, while unspecific RBPs are not enriched.^8^^,^^12^

### The SARS-CoV-2 RNA-protein interactome at subgenome resolution

In two replicate RAP-MS experiments, we found all 10 known components of the RMRP complex^12^^,^^14^ in purifications of RMRP RNA (Figure 1D) and 7 known subunits met our significance cutoff (adjusted p < 0.05, Table S1), confirming that our approach accurately captures physiological interactions of endogenous RNA in intact infected cells.

In SARS-CoV-2 RAP-MS experiments, we found a total of 254 significantly enriched proteins across all RNA purifications, of which 121, 244, and 202 were significantly enriched in gRNA, sgmRNA, and all viral RNA purifications, respectively (Table S1). The vast majority of these proteins (∼86%) were annotated as RBPs in a gene ontology (GO) enrichment analysis (Table S2). Our subgenome-resolved RNA interactome expands the number of direct SARS-CoV-2 RNA binders^7^^,^^8^^,^^9^ by almost 2-fold and represents the most comprehensive collection of host RBPs directly bound to SARS-CoV-2 RNA available.

We next analyzed the enrichment of SARS-CoV-2 proteins, of which 8 were detected in our subgenome-resolved RNA interactome. The structural proteins N, S, and M were most strongly enriched in gRNA purifications, while the host shutoff protein NSP1^15^ showed higher enrichment in sgmRNA purifications (Figure 1D; Table S1). This binding pattern is consistent with N binding to the RNA genome before it is packaged into assembling virions. There, gRNA is in close proximity to S and M, which likely facilitates RNA crosslinking.^16^

Analyzing host proteins identified in the three different RNA purifications (gRNA, sgmRNA, all viral RNA), we generally observed a large overlap (Figure S1B; Table S2). A GO term analysis confirmed enrichment of similar biological pathways for all three RNA interactomes (Table S2). The largest number of candidates were identified in sgmRNA purifications, which is consistent with their high intracellular abundance.^17^ We compared our subgenome-resolved SARS-CoV-2 RNA interactome with two other UV-crosslinking-based RNA interactomes.^7^^,^^9^ Notably, 44 proteins were jointly identified as direct SARS-CoV-2 RNA binders (Figure S1B), despite differences in methods, cell lines, and infection conditions.

### A consensus network of subgenome-resolved SARS-CoV-2 RNA interactions

To gain a deeper understanding of RNA interactions in cells of different organs susceptible to SARS-CoV-2 infection, we recorded a subgenome-resolved RNA interactome in human lung epithelial cells (A549^ACE2^)^18^ in addition to liver-derived Huh-7 cells.^19^ We identified 296 significantly enriched human proteins across all RNA purifications. While the A549^ACE2^ RNA interactome did not reach the depth of the Huh-7 RNA interactome (Table S1), we found 83 shared proteins among significantly enriched factors (Table S2), which exceeds the overlap observed with other UV-based RNA interactomes (Figure S1B).

Using the subgenome-resolved RNA interactomes together with our previously published data,^8^ we defined the consensus SARS-CoV-2 RNA interactome as those proteins enriched in replicate purifications of at least one RNA type (gRNA, sgmRNA, all viral RNA) in Huh-7 and A549^ACE2^ cells and passing a false discovery rate (FDR) cutoff of less than 10% (Table S2). To examine the connectivity between identified RBPs, we constructed a protein-protein association network using the consensus RNA interactome. In this network, each node is represented by a pie chart depicting the enrichment of each protein in gRNA, sgmRNA, and all viral RNA purifications in Huh-7 cells (Figure 1E). The enrichment for physical interactions between these proteins (Figure S1C; STAR Methods; p < 3.51 × 10^−65^), suggests that our RNA-protein interactome captures interconnected RBP-centric regulatory processes in infected cells.

### Several host RBPs preferentially bind sgmRNAs

Next, we directly compared the protein content of sgmRNA purifications to the protein content of gRNA purifications and derived enrichment and significance estimates across experiments in Huh-7 and A549^ACE2^ cells (Table S1). While annotated RBPs were strongly enriched in both gRNA and sgmRNA purifications, eukaryotic translation initiation factors displayed stronger enrichment in sgmRNA purifications relative to gRNA purifications (Figure 1F). Notably, several RBPs of the La-related protein (LARP) family, namely LARP1, LARP4, and LARP4B, were among the most strongly enriched sgmRNA-binding factors in both cell types (Figure 1F). Preferential binding of LARPs to sgmRNAs was not previously demonstrated, but is in line with the role of LARP1 in suppressing SARS-CoV-2 replication described in earlier works.^8^^,^^9^

Among sgmRNA binders (Figure 1F), SND1 stood out due to the presence of multiple putative nuclease domains, a feature that may indicate an involvement in RNA biogenesis or processing. SND1 contains five domains with homology to *Staphylococcus aureus* nuclease (SNase)^20^ in addition to a Tudor domain, which is known to bind dimethylated arginines and to facilitate the assembly of protein complexes.^21^^,^^22^^,^^23^^,^^24^^,^^25^ SNase domains in human SND1 appear to lack residues essential for nuclease activity and may instead mediate RNA binding.^20^ SND1 displays 98% amino acid identity between humans and *Rhinolophus ferrumequinum*, a natural host of SARS-like coronaviruses. While the precise role of SND1 in cellular RNA metabolism remains enigmatic,^26^ SND1 was the first component of the RNA-induced silencing complex (RISC) with a putative nuclease activity to be identified.^27^ However, SND1 does not have a role in site-specific mRNA cleavage.^28^^,^^29^^,^^30^ Instead, SND1 contributes to microRNA decay^31^^,^^32^ and degrades hyper-edited duplex RNAs.^33^ Previous studies that reported interactions between SND1 and RNA viruses described *in vitro* associations of synthetic RNA fragments in cellular extracts,^34^^,^^35^ or identified SND1 in global interactome datasets, lacking a detailed characterization of its function in viral RNA metabolism.^7^^,^^8^^,^^9^^,^^36^^,^^37^ Hence, we focused on dissecting the role of SND1 in SARS-CoV-2 infection.

### SND1 is a SARS-CoV-2 host dependency factor in human cells

**Figure 2:**
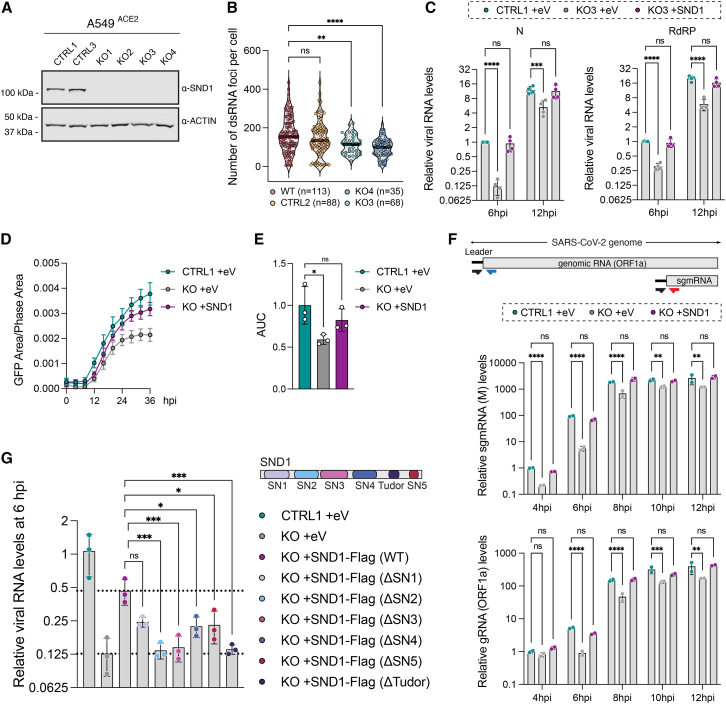
SND1 is a SARS-CoV-2 host dependency factor (A) Western blot analysis of SND1 knockout (KO) and control (CTRL) cell lines (A549^ACE2^). Actin serves as control. (B) Analysis of dsRNA foci in SND1 KO and CTRL cells compared to wild-type (WT) cells at 8 hpi. Median value indicated by black line. Representative images are shown in Figure S2B. p values determined by one-way ANOVA. (C) RT-qPCR of SARS-CoV-2 RNA levels in SND1 KO and CTRL cells transduced with empty vehicle (+eV) or SND1 (+SND1) as indicated. Quantification relative to 18S rRNA and CTRL at 6 hpi. Values are mean ± SD (n = 4 independent experiments; 3 technical replicates each). p values determined by two-way ANOVA with Dunnett’s test. (D) Time course of SARS-CoV-2-GFP growth in SND1 KO and CTRL cells transduced with empty vehicle (+eV) or SND1 (+SND1) as indicated. Values are mean ± SEM. Representative of 3 independent experiments is shown. (E) Area under the curve (AUC) analysis of 3 independent time course measurements of SARS-CoV-2-GFP growth. Normalization relative to mean of control (CTRL1 +eV). Values are mean ± SD. p values determined by one-way ANOVA with Dunnett’s test. (F) RT-qPCR of M (top) and ORF1a (bottom) RNA levels at 4–12 hpi in SND1 KO and CTRL cells transduced with empty vehicle (+eV), or SND1 (+SND1) as indicated. Quantification relative to 18S rRNA and CTRL at 4 hpi. Values are mean ± SD (n = 2 independent infections). Schematic: primer design to quantify gRNA and sgmRNA expression. p values determined by two-way ANOVA with Dunnett’s test. (G) RT-qPCR of SARS-CoV-2 RNA levels (N) in SND1 KO and CTRL cells transduced with empty vehicle (eV), full-length SND1 (WT), or SND1 deletion mutants as indicated. Quantification relative to 18S rRNA and CTRL. Values are mean ± SD (n = 3 independent infections). p values determined by two-way ANOVA with Dunnett’s test. ^∗∗∗∗^p < 0.0001, ^∗∗∗^p < 0.001, ^∗∗^p < 0.01, ^∗^p < 0.05, ns = not significant. All SARS-CoV-2 infections were performed in A549^ACE2^ cells at MOI 3 plaque-forming unit (PFU)/cell. See also Figure S2.

**Figure S2:**
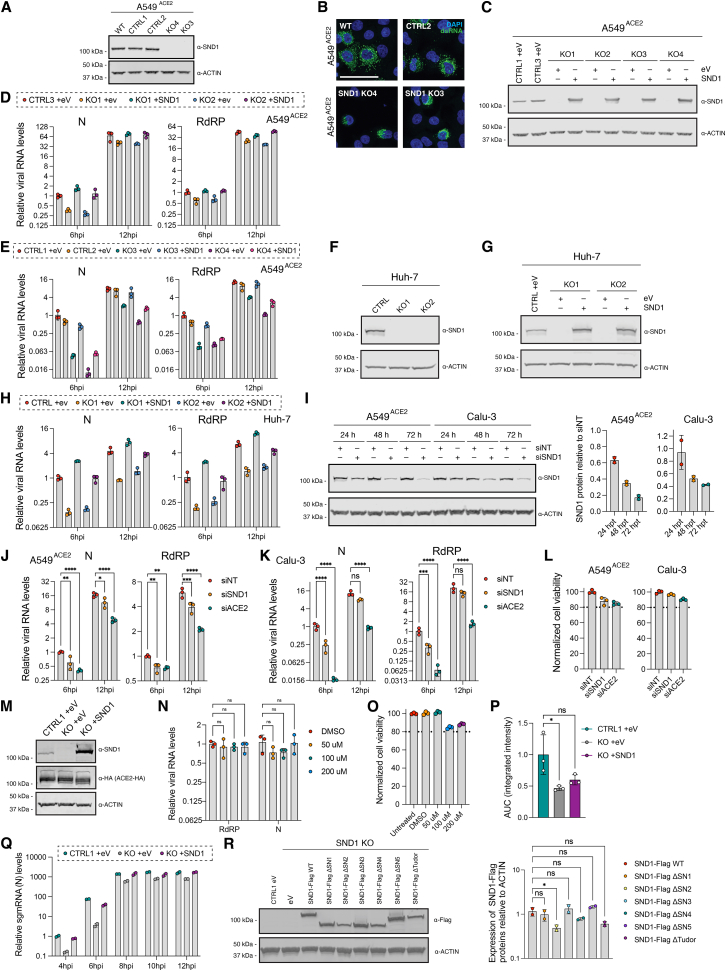
SND1 is a SARS-CoV-2 host factor, related to Figure 2 (A) Western blot analysis of SND1 knockout (KO), control (CTRL), and wild-type (WT) A549^ACE2^ cell lines. Expression of SND1 was evaluated relative to actin. (B) Representative images of IF staining for dsRNA (J2 antibody) using HCR detection in SARS-CoV-2 infected A549^ACE2^ cells at 8 hpi (MOI = 3 PFU/cell). Scale bars, 50 μm. (C) Western blot analysis of SND1 KO and CTRL cell lines (A549^ACE2^) transduced with empty vehicle (+eV), as well as SND1 KO cells transduced with SND1 (+SND1). Expression of SND1 was evaluated relative to actin. (D) RT-qPCR of SARS-CoV-2 RNA levels (left: N, right: RdRP) at 6 and 12 hpi in SND1 KO and CTRL cells transduced with empty vehicle (+eV) or SND1 (+SND1) as indicated. Two knockout clones shown in Figure 2A were analyzed. A549^ACE2^ cells were infected at MOI 3 PFU/cell. Quantification relative to 18S rRNA and CTRL at 6 hpi. Values are mean ± SD (n = 3 independent infections). (E) As in (D), but for two additional knockout clones shown in Figures 2A and S2A. (F) Western blot analysis of SND1 KO and CTRL cell lines (Huh-7). Expression of SND1 was evaluated relative to actin. (G) Western blot analysis of SND1 KO and CTRL cell lines (Huh-7), transduced with empty vehicle (+eV), as well as SND1 KO cells transduced with SND1 (+SND1). Expression of SND1 was evaluated relative to actin. (H) RT-qPCR of SARS-CoV-2 RNA levels (left: N, right: RdRP) at 6 and 12 hpi in SND1 KO and CTRL cells transduced with empty vehicle (+eV) or SND1 (+SND1) as indicated. Huh-7 cells were infected at MOI 3 PFU/cell. Quantification relative to 18S rRNA and CTRL at 6 hpi. Values are mean ± SD (n = 3 independent infections). (I) Western blot analysis of SND1 siRNA knockdown time course in A549^ACE2^ and Calu-3 cells at 24, 48, and 72 h post transfection. Expression changes are evaluated relative to non-targeting control and compared to changes in actin. Quantification of SND1 protein levels in two replicate experiments in A549^ACE2^ and Calu-3 cells is shown on the right. Values are mean ± SD (n = 2). (J) RT-qPCR of SARS-CoV-2 RNA levels (left: N, right: RdRP) at 6 and 12 hpi in A549^ACE2^ cells treated with SND1 siRNAs for 72 h. Cells were infected at MOI 3 PFU/cell. Quantification relative to 18S rRNA and CTRL at 6 hpi. Values are mean ± SD (n = 3 independent infections). p values determined by two-way ANOVA with Dunnett’s test. (K) As in (J), but for Calu-3 cells. (L) Cell viability assay of SND1 siRNA knockdown experiments at 72 h post transfection. Values are mean ± SD (n = 3). (M) Western blot analysis of SND1 KO and CTRL cell lines (A549^ACE2^) transduced with empty vehicle (+eV) or a constitutive lentiviral SND1 expression construct (+SND1). Expression of SND1 and ACE2-HA were evaluated relative to actin. (N) RT-qPCR of SARS-CoV-2 RNA levels at 6 hpi (left: RdRP, right: N) in A549^ACE2^ cells treated with DMSO or different concentrations of 2′-deoxythymidine-3′,5′-bisphosphate (pdTp) and infected with SARS-CoV-2 at MOI 3 PFU/cell. Quantification relative to 18S rRNA and DMSO treated cells. Values are mean ± SD (n = 3 independent infections). p values determined by two-way ANOVA with Dunnett’s test. (O) Cell viability assay of inhibitor treated and untreated cells shown in (N). Values are mean ± SD (n = 3 independent treatments). (P) Integrated fluorescence intensity-based analysis of three independent time course measurements of SARS-CoV-2-GFP reporter virus growth shown in Figure 2E. Normalization relative to mean of control (CTRL1 +eV). Values are mean ± SD. p values determined by ordinary one-way ANOVA with Dunnett’s test. (Q) RT-qPCR of N sgmRNA levels at 4–12 hpi in SND1 KO and CTRL cells transduced with empty vehicle (+eV) or SND1 (+SND1) as indicated. A549^ACE2^ cells were infected with SARS-CoV-2 at MOI 3 PFU/cell. Quantification relative to 18S rRNA and CTRL at 4 hpi. Primers to specifically quantify gRNA and sgmRNA expression levels (Figure 2F) were used. Values are mean ± SD (n = 2 independent infections). (R) Western blot analysis of SND1 KO and CTRL cells transduced with empty vehicle (eV) or a constitutive lentiviral expression construct encoding the indicated SND1 proteins. Expression of FLAG-tagged SND1 proteins was quantified in duplicates and evaluated relative to actin (right). Values are mean ± SD. p values determined by ordinary one-way ANOVA with Dunnett’s test. ^∗∗∗∗^p < 0.0001, ^∗∗∗^p < 0.001, ^∗∗^p < 0.01, ^∗^p < 0.05, ns = not significant.

We generated four clonal SND1 knockout (KO) cell lines as well as matched control cell lines using Cas9 and single-guide RNAs in A549^ACE2^ cells and validated complete loss of SND1 protein expression in KO cells (Figures 2A and S2A). Upon infection of SND1 KO cells with SARS-CoV-2, we noted reduced double-stranded RNA (dsRNA) accumulation (Figures 2B and S2B) and diminished viral RNA replication (Figure 2C) compared to control cells that was most pronounced at early infection time points. Complementation of SND1 by exogenous expression in KO cells (Figure S2C) led to a complete rescue of the KO phenotype in three KO clones (Figures 2C, S2D, and S2E), while a partial rescue was observed in one KO clone (Figure S2E). To corroborate this phenotype in a different cell type, we generated two additional SND1 KO clones in Huh-7 cells (Figure S2F) and performed SARS-CoV-2 infection experiments with and without SND1 complementation (Figure S2G). Again, SARS-CoV-2 replication was impaired in SND1 KO cells and exogenous expression of SND1 led to a complete rescue (Figure S2H). Transient depletion of SND1 proteins by siRNA knockdown (Figure S2I) also led to a significant reduction of SARS-CoV-2 RNA replication in two different cell types (Figures S2J–S2L). We concluded that the reduction in SARS-CoV-2 RNA replication was due to loss of SND1 protein expression and selected a single SND1 KO clone in A549^ACE2^ cells for all subsequent experiments. No difference in ACE2 expression was observed between this KO clone and a matched control clone, including after transduction with empty vehicle or a lentiviral SND1 expression construct (Figure S2M). Inhibition of the putative nuclease activity of SND1 with 2′-deoxythymidine-3′,5′-bisphosphate (pdTp), a competitive inhibitor of staphylococcal nucleases,^38^ at concentrations reported to inhibit SND1 nuclease activity in human cells,^39^ did not affect SARS-CoV-2 replication (Figures S2N and S2O). Thus, a putative nuclease activity of SND1 is dispensable for its function in SARS-CoV-2 infection.

To confirm that loss of viral RNA replication led to a reduction in virus growth over multiple infection cycles, we used fluorescent SARS-CoV-2-GFP virus^40^ and recorded viral growth kinetics for 36 h. We observed delayed viral growth early during the infection cycle (0–16 hpi), as well as a reduced production of SARS-CoV-2-GFP virus in SND1 KO cells (Figures 2D, 2E, and S2P). Importantly, the viral growth defect could be reversed by exogenous SND1 expression in KO cells (Figures 2D and 2E). To resolve how SND1 depletion affects viral RNA production, we measured changes in SARS-CoV-2 gRNA and sgmRNA levels in 2-h intervals, early during the infection cycle. To quantify expression changes in gRNA and sgmRNA separately, we designed reverse-transcriptase quantitative PCR (RT-qPCR) primers spanning the leader-body junction of two sgmRNAs (M and N) and the leader-ORF1a junction (Figure 2F). Starting at 4 hpi, the sgmRNAs M and N were depleted in SND1 KO cells compared to control cells (Figures 2F, top and S2Q). Depletion of sgmRNAs was most pronounced at 6 hpi and persisted at subsequent infection time points. Exogenous SND1 expression reversed this phenotype (Figures 2F, top and S2Q). Interestingly, we noted a delayed effect of SND1 depletion on gRNA expression levels. At 4 hpi, we observed comparable levels of gRNA in SND1 KO, control, and rescue cells, confirming that cells were infected with similar efficiencies (Figure 2F, bottom). At 6 hpi, when productive replication of incoming gRNA is underway, SND1 KO cells displayed a significant reduction in gRNA levels that was observed at all subsequent time points and was reversed by SND1 complementation (Figure 2F, bottom). These data demonstrate that SND1 is a host factor promoting SARS-CoV-2 replication and virus growth in human cells.

### The SNase 3 domain is required for SND1 function in SARS-CoV-2 infection

Next, we investigated whether deleting individual protein domains impacts the function of SND1 in SARS-CoV-2 infection. We complemented SND1 KO cells with C-terminally tagged full-length SND1 or SND1 deletion mutants (Figures 2G and S2R). Deletion of SNase 1, 3, and 5 did not alter protein expression levels compared to full-length SND1 (Figure S2R). While full-length SND1 could reverse the loss of viral RNA replication in SND1 KO cells, proteins lacking SNase 3 displayed no rescue activity (Figure 2G). Deletion of SNase 1, 4, and 5 only had a modest effect on the rescue capacity of SND1 (Figure 2G). For Tudor and SNase 2-deleted proteins, which displayed no rescue activity, we cannot exclude that their reduced expression (Figure S2R) may contribute to the lack of rescue (Figure 2G). Together, these data suggest that the SNase 3 domain is critical for SND1 function in the context of SARS-CoV-2 infection.

### SND1 binds negative-sense viral RNA

**Figure 3:**
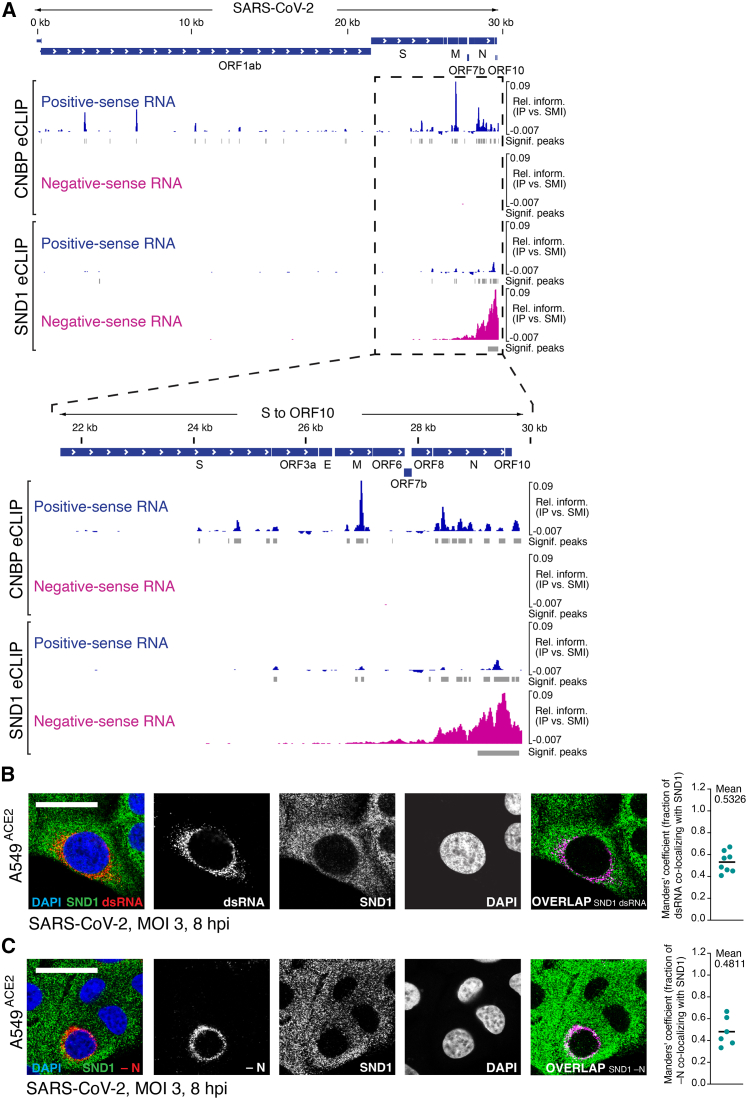
SND1 binds negative-sense viral RNA (A) Alignment of strand-separated eCLIP data for SND1 and CNBP^8^ to SARS-CoV-2 genome. Relative information in IP vs. size-matched input (SMI) is calculated at each position (STAR Methods) and displayed for positive-sense (blue) and negative-sense RNA (magenta). Peaks significantly enriched relative to SMI are indicated in gray. Lower panel displays zoom in to the S to ORF10 region. eCLIP was performed in Huh-7 cells at 24 hpi. (B) IF staining of SND1 and dsRNA in SARS-CoV-2 infected A549^ACE2^ cells at 8 hpi (MOI = 3 PFU/cell). Representative images are shown. Overlap is quantified by Manders’ co-localization coefficient (n = 8 images). Uninfected cells shown in Figure S3A. (C) HCR RNA-FISH for negative-sense RNA (−N) combined with HCR IF for SND1 in SARS-CoV-2 infected A549^ACE2^ cells at 8 hpi (MOI = 3 PFU/cell). Representative images are shown. Overlap is quantified by Manders’ co-localization coefficient (n = 6 images). Cells were denatured prior to −N RNA detection; non-denatured cells shown in Figure S3B. Scale bars, 25 μm. See also Figure S3 and Table S3.

Next, we mapped direct SND1 binding sites on SARS-CoV-2 RNA using enhanced crosslinking and immunoprecipitation (eCLIP).^41^ Unexpectedly, we observed a strong preference of SND1 for binding negative-sense RNA at the 3′ end of the viral genome (Figure 3A). This binding pattern is unusual given the excess of positive-sense over negative-sense viral RNA in infected cells.^17^ Indeed, when analyzing the binding pattern of cellular nucleic acid-binding protein (CNBP), another sgmRNA binder (Table S1), we observed near-exclusive binding to positive-sense RNA (Figure 3A). Binding of SND1 to negative-sense RNA was particularly striking across N and increased toward the 3′ end of N, extending through ORF10 to the 3′ UTR (Figure 3A). We found that 84% of all sequence reads in N and 91% of all reads in ORF10 originated from the negative strand (Table S3), which is a significant enrichment compared to the positive strand (adjusted p < 4.9 × 10^−324^; STAR Methods) and suggests selective recognition of negative-sense RNA. Since negative-sense RNA intermediates are templates for SARS-CoV-2 RNA transcription and genome replication, the binding pattern of SND1 points to a role in SARS-CoV-2 RNA synthesis.

**Figure S3:**
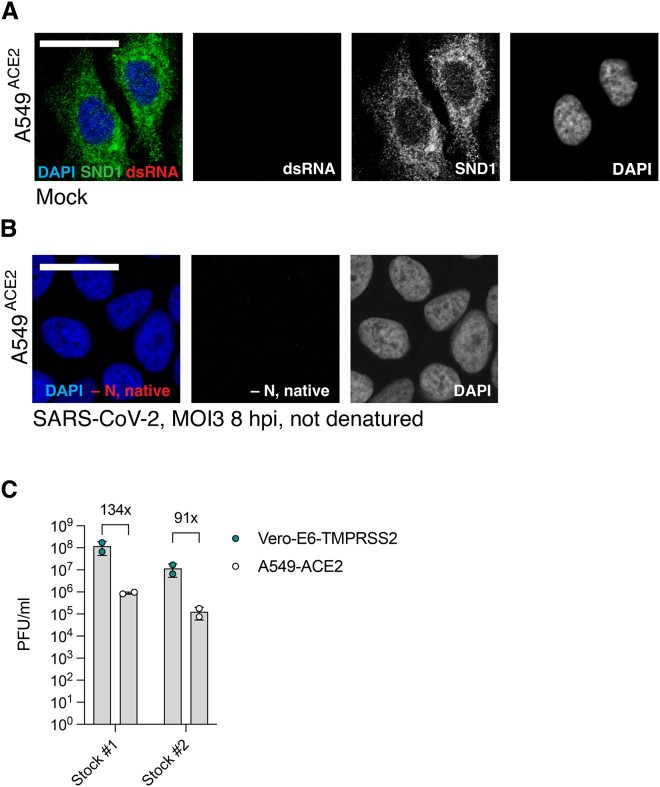
Subcellular localization of SND1 and viral replication products, related to Figure 3 (A) IF staining of SND1 and dsRNA (J2 antibody) in uninfected A549^ACE2^ cells at 8 hpi. (B) HCR RNA-FISH for negative-sense RNA of the N gene (−N) in SARS-CoV-2 infected A549^ACE2^ cells without denaturation prior to probe hybridization. Scale bars, 25 μm. (C) Titers of virus stocks determined by plaque assays on Vero-E6-TMPRSS2 and A549^ACE2^ cells. Values are mean ± SD (n = 2).

To observe interactions between SND1 and SARS-CoV-2 RNA replication products with subcellular resolution, we visualized SND1 and dsRNA by immunofluorescence (IF) microscopy. SND1 formed homogenously distributed foci in the cytoplasm (Figure 3B). dsRNA accumulated in the perinuclear region and SND1 foci were interspaced with dsRNA foci that partially overlapped in this area (Figures 3B and S3). We quantified the co-localization between dsRNA and SND1 using Manders’ co-localization coefficient^42^ and found that on average ∼53% of dsRNA foci overlapped with SND1 (Figure 3B). We used hybridization chain reaction (HCR) to combine IF microscopy with RNA fluorescence *in situ* hybridization (RNA-FISH) and simultaneously visualized SND1 together with negative-sense viral RNA.^43^ Similar to dsRNA, we observed an interspaced and partially overlapping localization pattern between SND1 foci and the negative-sense RNA of N (Figure 3C). On average ∼48% of negative-sense RNA foci overlapped with SND1 (Figure 3C). Together, our imaging analysis is consistent with SND1 localizing to the proximity of dsRNA-containing RTCs (Figure 3B), where SND1 directly interacts with SARS-CoV-2 negative-sense RNA (Figures 3A and 3C).

### SND1 is required for nascent viral RNA synthesis

**Figure 4:**
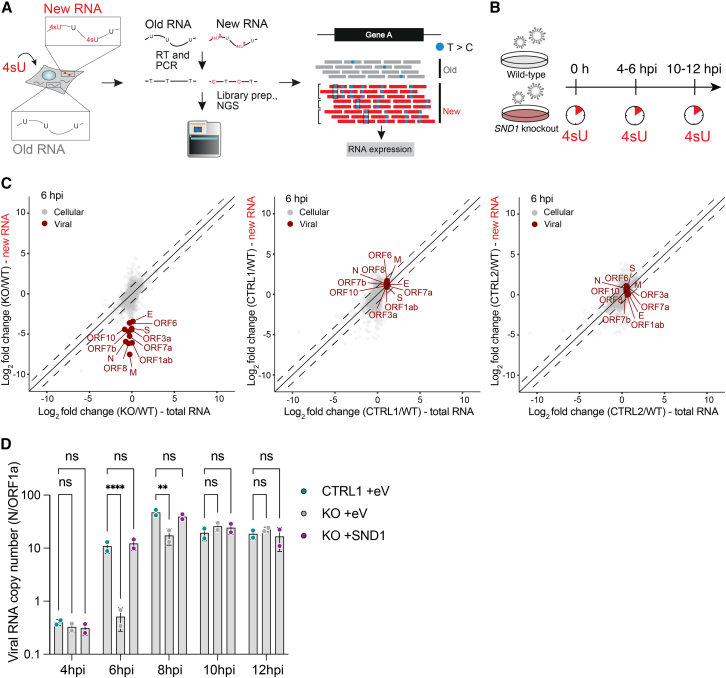
SND1 is required for nascent SARS-CoV-2 RNA synthesis early during infection (A) Outline of SLAM-seq method. (B) Strategy to measure nascent SARS-CoV-2 RNA synthesis in SND1 knockout (KO) and control (CTRL) or wild-type (WT) cells. (C) SLAM-seq analysis at 6 hpi. Average log_2_ fold changes in total RNA (x axis) and newly synthesized RNA (y axis) are shown for SND1 KO and CTRL cells, relative to WT cells (n = 2 independent infections). (D) Absolute quantification of viral RNA copy numbers by digital droplet PCR. Ratio of sgmRNA (N) relative to gRNA (ORF1a) is shown at 4–12 hpi, in SND1 KO and CTRL cells transduced with empty vehicle (+eV) or SND1 (+SND1) as indicated. Cells infected with SARS-CoV-2 at MOI 3 PFU/cell. Values are mean ± SD (n = 2 independent infections). p values determined by two-way ANOVA with Dunnett’s test. ^∗∗∗∗^p < 0.0001, ^∗∗^p < 0.01, ns = not significant. See also Figure S4.

**Figure S4:**
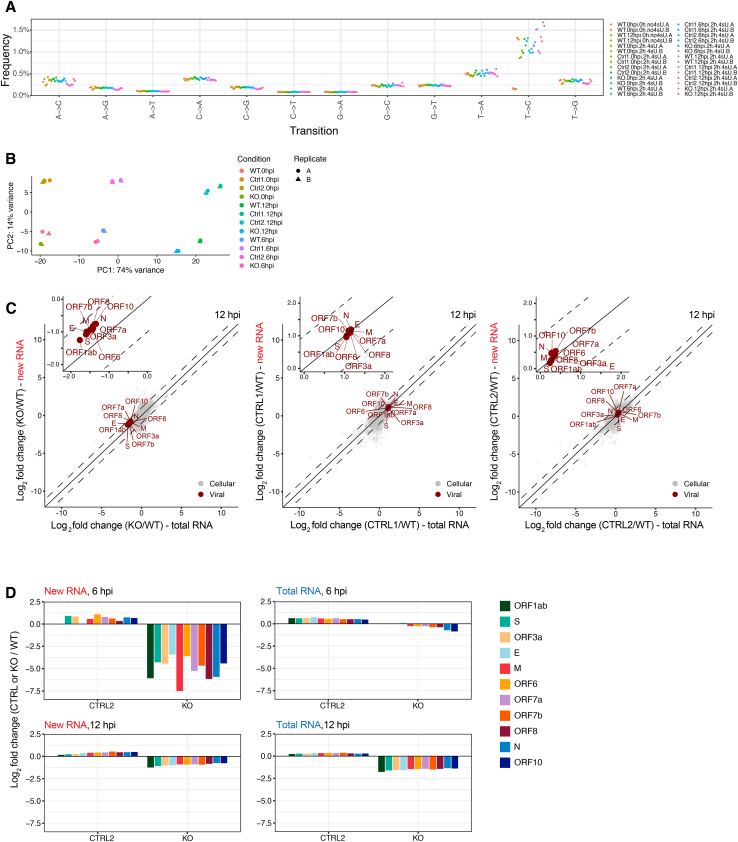
Measuring changes in viral RNA synthesis with SLAM-seq, related to Figure 4 (A) Mismatch frequencies observed in SLAM-seq libraries. (B) Principal component analysis of SLAM-seq experiments. (C) Analysis of SLAM-seq experiments at 12 hpi using GRAND-SLAM. Scatter plot of average log_2_ fold changes in total RNA (x axis) and newly synthesized RNA (y axis) is shown for SND1 knockout (KO) and control (CTRL) cells, relative to wild-type (WT) cells (n = 2 independent experiments). Insets show a zoom-in view on viral genes for improved visibility. (D) Bar graph visualization of log_2_ fold changes for new RNA (left) and total RNA (right) observed in SLAM-seq experiments shown in (C) and Figure 4C for viral genes at 6 (top) and 12 hpi (bottom).

To quantitatively assess the effect of SND1 depletion on newly synthesized and pre-existing viral RNA we used the thiol-(SH)-linked alkylation of RNA for metabolic labelling sequencing (SLAM-seq) approach^44^ (Figure 4A). We metabolically labeled A549^ACE2^ cells with 4-thiouridine (4sU) at 0 (mock), 4–6, and 10–12 hpi in wild-type (WT), SND1 KO, and control cells (Figure 4B). We observed T-to-C transitions, the characteristic signature of 4sU incorporation into nascent RNA, only in 4sU-labeled cells (Figure S4A) and samples clustered according to their genotype and infection time point (Figure S4B). Analysis of SLAM-seq data using GRAND-SLAM^45^ revealed a dramatic loss of all newly synthesized viral RNAs in SND1 KO cells, but not in control cells at 6 hpi (Figures 4C and S4D). Strikingly, we did not observe a concomitant reduction in total viral RNA (Figures 4C and S4D), indicating that the synthesis of nascent viral RNA is impaired as a result of SND1 depletion. At 12 hpi, we observed only a mild reduction in newly synthesized viral RNA levels, paralleled by a more pronounced decrease in total viral RNA (Figures S4C and S4D). Notably, the exponential phase of viral RNA synthesis occurs 3–6 h after infection,^46^^,^^47^ matching the time window in which we observe RNA synthesis defects in SND1-depleted cells. Together, these data demonstrate that SND1 functions at an early step of viral RNA synthesis.

Since SARS-CoV-2 transcription predominantly produces sgmRNAs,^17^ we speculated that a loss of nascent viral RNA synthesis would lead to a reduced accumulation of sgmRNA relative to gRNA. Indeed, when measuring absolute copy numbers of sgmRNA (N) and gRNA (ORF1a) by digital droplet PCR (ddPCR), we observed a significant reduction in sgmRNA levels relative to gRNA in SND1 KO cells at 6 and 8 hpi (Figure 4D), when nascent RNA synthesis is impaired (Figure 4C). SND1 complementation in KO cells restored the ratio of sgmRNA relative to gRNA back to levels observed in control cells (Figure 4D). At later infection time points, gRNA and sgmRNA are not differentially affected by SND1 KO (Figure 4D). These data are consistent with the loss of SND1 impairing nascent viral RNA synthesis, which perturbs not only the abundance of all viral RNAs, but also the pattern of viral gene expression. Together with Figure 2F, these data confirm that the replication defect in SND1 KO cells occurs at the level of viral RNA synthesis and cannot be explained by differences in incoming virus genomes.

### SND1 interacts with RTC components involved in viral RNA biogenesis

**Figure 5:**
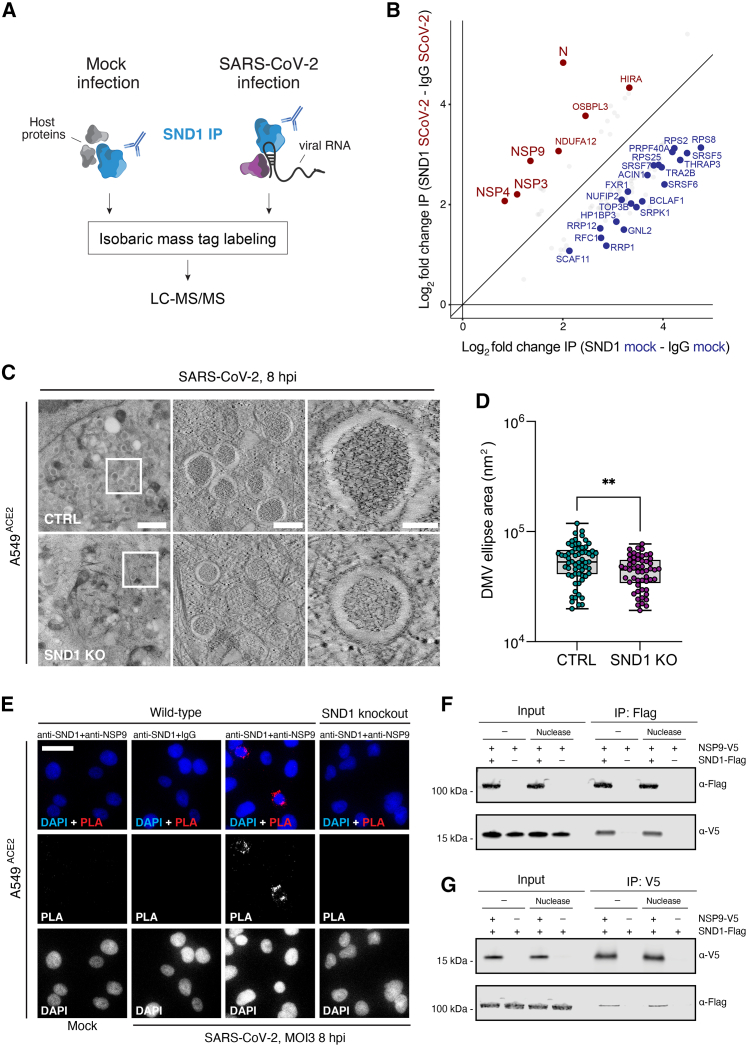
SND1 interacts with RTC components involved in viral RNA biogenesis (A) Strategy to globally identify SND1 protein-protein interactome changes upon SARS-CoV-2 infection. (B) Fold change correlation plot displaying SND1 interacting proteins enriched over IgG control in SARS-CoV-2 infected (SCoV-2, y axis) and uninfected (mock, x axis) A549^ACE2^ cells (n = 2 independent experiments). Candidates with a fold change > 1.5 or < 0.66 and FDR < 0.2 in infected relative to uninfected cells are displayed. Proteins with a substantial SND1 interaction change (absolute log_2_ fold change > 1, FDR < 0.05) are highlighted in red and blue. (C) Computational slices of representative electron tomograms of SND1 knockout (KO) and control (CTRL) cells infected with SARS-CoV-2. Zoom in to region containing DMVs is shown at different magnifications. Left: scale bars, 1 μm; center: scale bars, 250 nm; right: scale bars, 100 nm. (D) Quantification of cross-sectional DMV area in SND1 KO and CTRL cells. Box: 25^th^ and 75^th^ percentiles. Whiskers: minimum to maximum. Median indicated by line. p value determined by t test, ^∗∗^p < 0.01. (E) Proximity ligation assay for SND1 and NSP9. Mock-infected cells are compared to SARS-CoV-2 infected cells. Scale bars, 30 μm. (F) CoIP western blot analysis for epitope-tagged SND1 and NSP9 proteins expressed in uninfected HEK293T cells with and without nuclease (benzonase) treatment. SND1-FLAG served as bait. Input lysates are shown on the left. (G) As in (F), but using NSP9-V5 as bait. See also Figure S5 and Table S4.

**Figure S5:**
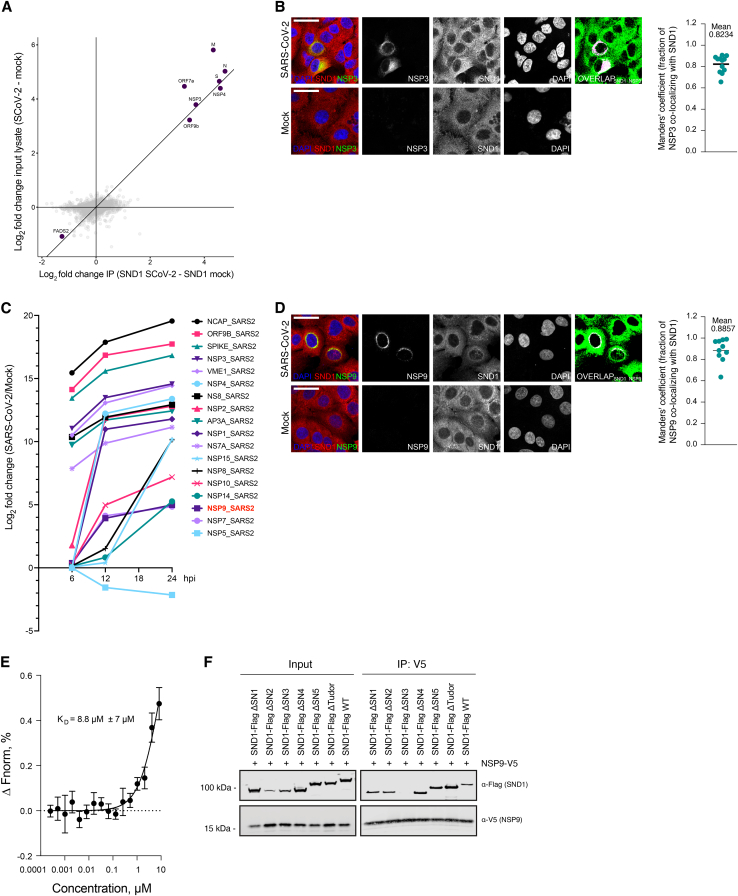
Expression, localization, and interaction analyses for virus and host proteins, related to Figure 5 (A) Scatter plot displaying average log_2_ fold changes in SND1 coIP experiments (x axis) and average log_2_ fold changes in total proteome measurements of input lysates used for coIP experiments (y axis). SARS-CoV-2 infected cells are compared relative to uninfected cells in coIPs and total proteome measurements (n = 2 independent infections). Proteins that display a significant interaction and expression change (absolute log_2_ fold change > 1, FDR < 0.05) upon SARS-CoV-2 infection cells are highlighted. (B) IF staining of SND1 and NSP3 proteins in SARS-CoV-2 infected A549^ACE2^ cells at 8 hpi (MOI = 10 PFU/cell). Mock-infected cells were stained as controls. Representative images are shown. Overlap between NSP3 and SND1 is quantified by Manders’ co-localization coefficient (right, n = 12 images). Scale bars, 30 μm. (C) Log_2_ fold changes for viral proteins observed in total proteome measurements of SARS-CoV-2 infected A549^ACE2^ cells at different infection time points, as previously reported.^18^ (D) As in (B), but for SND1 and NSP9. Overlap between NSP9 and SND1 is quantified by Manders’ co-localization coefficient (right, n = 10 images). (E) Microscale thermophoresis (MST) assay to monitor binding of recombinant NSP9 protein to recombinant SND1 protein *in vitro*. Unlabeled NSP9 protein (254 pM to 8.33 μM) was titrated against site-specific cysteine-labeled SND1 (10 nM) and thermophoresis was recorded. Change in fluorescence (ΔFnorm) was measured at MST on-time of 10 s. Values are mean ± SD (n = 3 independent measurements). (F) CoIP western blot analysis for epitope-tagged SND1 and NSP9 proteins exogenously expressed in uninfected HEK293T cells. SND1-FLAG constructs are deleted for indicated protein domains. NSP9-V5 served as bait. Input lysates are shown on the left. Anti-FLAG and anti-V5 antibodies are used to detect tagged SND1 and NSP9 proteins, respectively. SN, SNase.

Next, we characterized the SND1 protein-protein interactome in SARS-CoV-2 infected and uninfected cells using co-immunoprecipitation and quantitative mass spectrometry (coIP MS) (Figure 5A). We normalized SND1 interacting proteins to an unspecific control (IgG) and confirmed enrichment of SND1, along with known binding partners, such as metadherin (MTDH)^48^ (Table S4). Next, we determined which SND1 interactors were significantly changed in infected and uninfected cells (Figure 5B; Table S4; STAR Methods). When focusing on interactions weakened upon infection, we found multiple host RBPs and an overrepresentation of pathways linked to mRNA processing (Table S4). Interactions gained upon infection were dominated by viral proteins (N, NSP3, NSP4, NSP9) and several host factors associated with membranes (OSBPL3,^49^ NDUFA12,^50^ NRP1,^51^ ABCF1,^52^ GJA1^53^). This likely reflects the localization of SND1 near membrane-associated RTCs (Figures 3B and 3C). Only a single host protein that displayed differential SND1 association had altered protein abundance levels when comparing SARS-CoV-2 infected to uninfected cells in a total proteome analysis of coIP input lysates (Figure S5A; Table S4). As expected, viral proteins, including 3 out of 4 SND1 interactors, were prominently detected in our total proteome. Several SND1-interacting viral proteins are involved in coronavirus RNA biogenesis and localize to DMVs. Specifically, NSP3 and NSP4 drive DMV biogenesis and constitute a molecular pore involved in exporting viral RNA.^54^^,^^55^ The two remaining SND1 interactors NSP9 and N promote coronavirus transcription and replication through direct RNA binding.^2^^,^^56^^,^^57^^,^^58^

### Loss of SND1 affects SARS-CoV-2 replication organelles

The association between SND1 and NSP3 and NSP4 could point to a contribution of alterations in SARS-CoV-2 DMVs to the SND1 KO phenotype. Indeed, SND1 and NSP3 co-localize in SARS-CoV-2 infected cells. On average ∼82% of NSP3 foci overlap with SND1 (Figure S5B). To test if SND1 affects SARS-CoV-2 DMVs, we employed high pressure freezing and freeze substitution (HPF-FS) and performed electron tomography of SND1 KO and control cells after SARS-CoV-2 infection. While DMV formation was readily observed in control and KO cells at 8 hpi, DMVs were smaller in SND1 KO cells as indicated by a significantly reduced cross-sectional DMV area (Figures 5C and 5D). The reduced DMV size in SND1 KO cells may be linked to reduced or delayed production of nascent viral RNA in the absence of SND1. Indeed, DMVs formed in the absence of viral RNA production are smaller compared to DMVs containing viral RNA.^55^

### SND1 forms a complex with the RNA biogenesis factor NSP9

We next wondered if other SND1-interacting viral proteins also contribute to the SND1 KO phenotype. NSP9 was the only viral SND1 interactor not detected in our total proteome, suggesting that NSP9 is not abundantly expressed. Indeed, a previous proteome analysis of the same A549^ACE2^ cell line^18^ revealed that NSP9 was among the least abundantly expressed SARS-CoV-2 proteins at all infection time points (Figure S5C). Hence, the interaction between NSP9 and SND1 is not driven by protein abundance and likely constitutes a specific interaction.

NSP9 is a single-stranded RBP that is covalently NMPylated at a conserved N-terminal asparagine by the nidovirus RdRP-associated nucleotidyltransferase (NiRAN) domain of NSP12.^59^^,^^60^ Since SARS-CoV-2 is thought to initiate RNA transcription starting with an NTP,^61^ NMPylated NSP9 may prime SARS-CoV-2 RNA synthesis.^59^ Moreover, NSP9 may also play a role in capping of SARS-CoV-2 RNA.^62^^,^^63^ Hence, we next focused on dissecting the SND1-NSP9 interaction.

Using the proximity ligation assay (PLA), we observed a specific SND1-NSP9 interaction only in infected cells and this interaction was not detectable in SND1 KO cells or when replacing one of the PLA antibodies with an unspecific control (Figure 5E). SND1 and NSP9 interact in the perinuclear region (Figure 5E), where viral RNAs are synthesized and RTC-associated DMVs are formed.^64^ Complementing PLA, we used IF microscopy to visualize both SND1 and NSP9. On average ∼89% of NSP9 foci overlapped with SND1 (Figure S5D). Next, we expressed epitope-tagged SND1 and NSP9 proteins in uninfected HEK293T cells and performed reciprocal coIP experiments. We observed a specific interaction between SND1 and NSP9 in the absence of any other viral components (Figure 5F). Importantly, the interaction was unaffected by nuclease treatment and was robustly detected using either SND1 or NSP9 as bait (Figures 5F and 5G). These data demonstrate that SARS-CoV-2 RNA or the RTC are not required to mediate the protein-protein interaction between SND1 and NSP9. Moreover, recombinant SND1 and NSP9 directly interact *in vitro* with a moderate affinity in the low micromolar range (Figure S5E). To test if deleting individual protein domains in SND1 altered the interaction with NSP9, we used the SND1 domain deletion constructs described above and performed coIP experiments with NSP9 as bait. When deleting the SNase 3 domain from SND1, we failed to detect an interaction with NSP9, suggesting that SNase 3 is required for the SND1-NSP9 interaction (Figure S5F). Since deletion of SNase 3 impairs SND1 function in SARS-CoV-2 infection (Figure 2G), the SND1-NSP9 interaction appears to be functionally relevant.

### Positive and negative-sense viral RNAs are covalently linked to NSP9 via their 5′ ends

**Figure 6:**
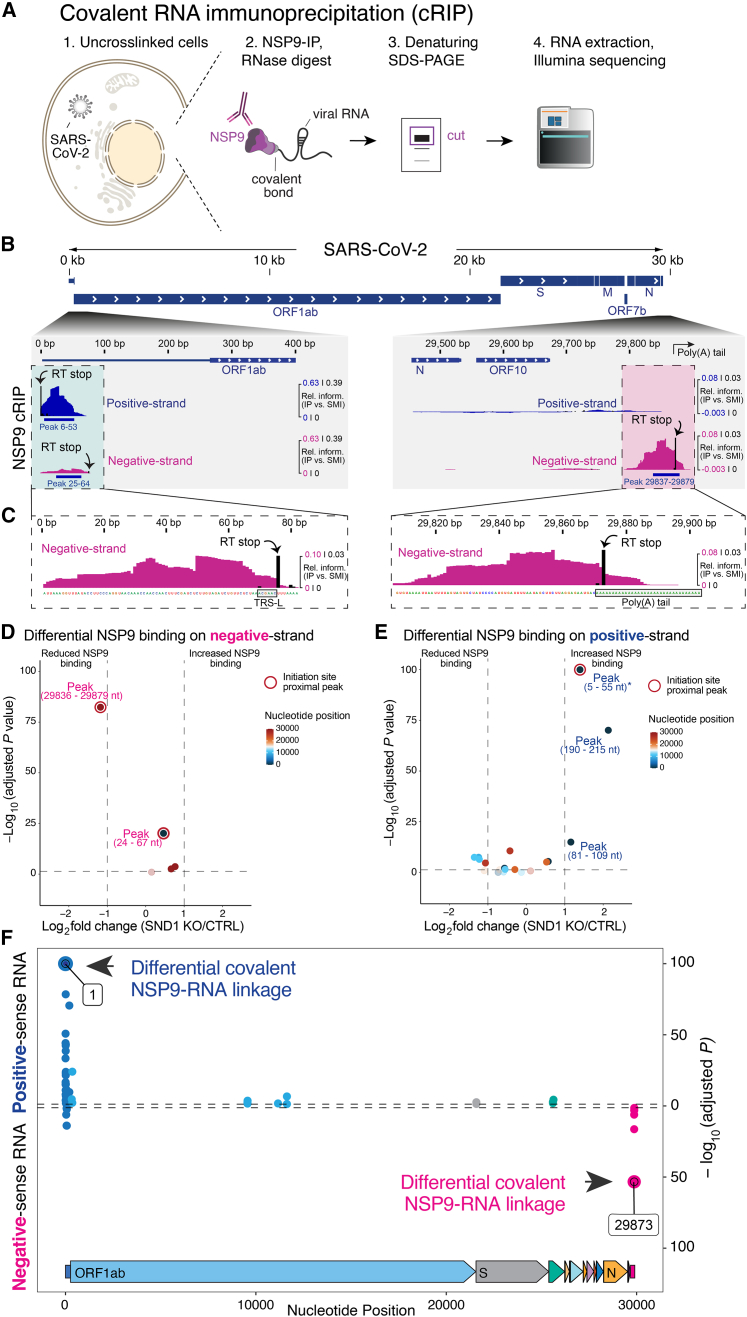
NSP9 is covalently linked to SARS-CoV-2 RNA and SND1 modulates NSP9 occupancy and its covalent linkage to viral RNA (A) Schematic of covalent RNA immunoprecipitation (cRIP) to map covalent RNA-protein linkages formed in the absence of UV-crosslinking. (B) Alignment of strand-separated NSP9 cRIP data to SARS-CoV-2 genome. Relative information in IP vs. SMI is calculated at each position (STAR Methods) and displayed for positive-sense (blue) and negative-sense (magenta) RNA. Zoom-in views of 5′ and the 3′ end of the viral RNA genome are shown for representative experiment in A549^ACE2^ cells at 8 hpi. NSP9 peaks significantly enriched relative to SMI (log_2_ fold change > 2, p < 0.05, one-sided Fisher test) are indicated. RT stops are denoted in black (relative information in IP vs. SMI). Green and red boxes indicate regions of interest. Scale: colored numbers relate to normalized coverage (IP vs. SMI); black numbers relate to normalized RT stops (IP vs. SMI). (C) Zoom-in view of NSP9 cRIP signal and RT stops (relative information in IP vs. SMI) in negative-sense RNA at 5′ and 3′ end of the viral genome. Gray boxes indicate TRS-L region and poly(A/U) tail. Sequence corresponds to positive strand. (D) SND1-dependent changes in NSP9 binding on peak regions relative to non-peak regions in negative-sense RNA in A549^ACE2^ cells (SND1 KO vs. CTRL) at 12 hpi. Color scale reflects localization of peaks. Peaks with strongest NSP9 binding change are annotated. Initiation site-proximal peaks are encircled. (E) As in (D) but for positive-sense RNA. ^∗^Actual p value of peak (5–55 nt) was computed as p < 4.9 × 10^−324^; for visualization this p value was set to p = 1.0 × 10^−100^. (F) SND1-dependent changes in covalent NSP9-RNA linkages across the SARS-CoV-2 genome in positive and negative-sense RNA. Representative experiment in A549^ACE2^ cells (SND1 KO vs. CTRL) at 12 hpi is shown. Actual p value of nucleotide 1 was computed as p < 4.9 × 10^−324^; for visualization this p value was set to p = 1.0 × 10^−100^. See also Figure S6 and Tables S3, S5, and S6.

We next explored the potential protein-priming function of NSP9^59^^,^^65^ in an authentic infection context. Since priming of viral RNA synthesis by NMPylated NSP9 would result in the covalent linkage of nascent RNA to NSP9 via its 5′ end, we devised a strategy to identify covalent linkages between NSP9 and RNA. When omitting UV-crosslinking, CLIP is ideally suited for mapping covalent protein-RNA linkages formed in cells. Importantly, the stringency of CLIP, which denatures immunopurified complexes in Laemmli buffer,^66^ circumvents recovery of non-covalent interactions.^67^^,^^68^^,^^69^ We performed eCLIP for NSP9 at 8 and 12 hpi without prior UV-crosslinking. To differentiate this approach from CLIP, we refer to it as covalent RNA immunoprecipitation (cRIP) (Figure 6A). We separated sequence reads covalently linked to NSP9 according to their origin from the positive or negative viral RNA strand and identified peaks significantly enriched relative to a size-matched input (SMI) control (STAR Methods). This analysis revealed that covalent binding of NSP9 to viral RNA predominantly occurs at the ends of the SARS-CoV-2 RNA genome (Table S5). At the 5′ end, we observed a strongly enriched NSP9 attachment site in positive-sense RNA covering the first ∼50 nucleotides of the genome (Figure 6B, green box). In this region 88% of all sequence reads originated from the positive strand at 8 hpi (adjusted p < 4.9 × 10^−324^, Table S3). This pattern reversed at the 3′ end of the SARS-CoV-2 genome. Here, we found a strongly enriched NSP9 attachment site in negative-sense RNA that is centered on the junction between the 3′ UTR and the poly(A) tail, where negative-sense RNA synthesis initiates (Figure 6B, red box). The majority of sequence reads in this region originated from the negative strand (74%) at 8 hpi (adjusted p < 9.58 × 10^−51^, Table S3). Preferential binding of NSP9 to negative-sense RNA is notable due to its limited abundance,^17^ indicating that the negative strand is the preferred substrate for NSP9 activity at the 3′ end of the genome.

**Figure S6:**
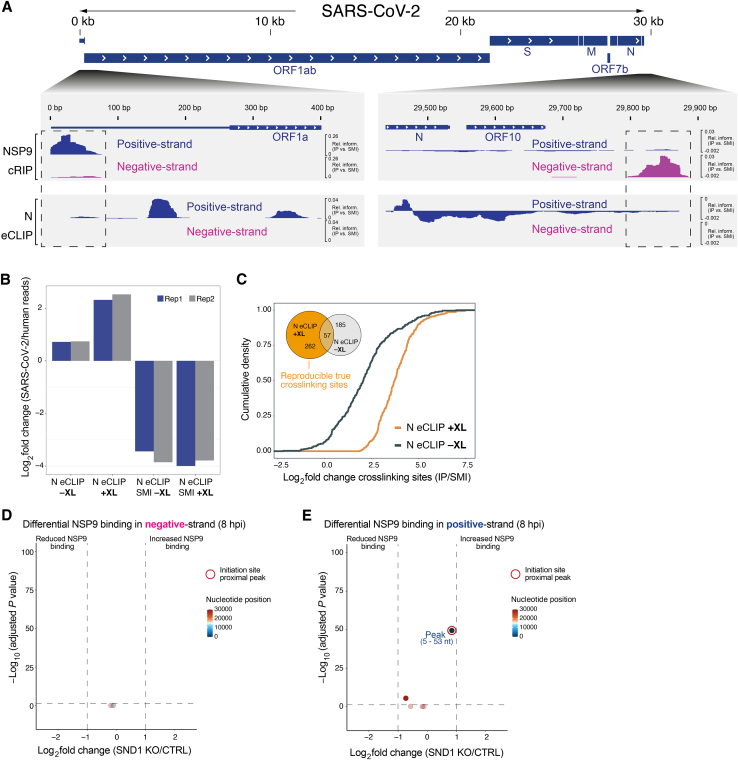
Analysis of N and NSP9 RNA binding on viral RNA, related to Figure 6 (A) Comparison of the viral RNA binding pattern observed for NSP9 (cRIP, top) and N (eCLIP, bottom) at 12 hpi in A549^ACE2^ cells. Alignment of strand-separated cRIP and eCLIP data to the SARS-CoV-2 RNA genome is shown. Relative information in IP vs. SMI is calculated at each position (STAR Methods) and displayed for positive-sense RNA (blue) and negative-sense RNA (magenta). Zoom-in views of the 5′ end (left) and the 3′ end (right) of the viral RNA genome are shown and regions of interest are highlighted by dashed box. (B) Enrichment of reads mapping to the SARS-CoV-2 genome relative to the human genome in N eCLIP experiments with (+XL) and without (−XL) UV-crosslinking, as well as corresponding SMI libraries. (C) Cumulative density function displaying enrichment of crosslinked nucleotides in N IP relative to SMI experiments with and without UV-crosslinking. Inset shows Venn diagram displaying the overlap of true crosslinking sites reproducibly found in N eCLIP experiments with sites found in N eCLIP experiments without UV-crosslinking. (D) Volcano plot displaying SND1-dependent changes in NSP9 binding on peak regions relative to non-peak regions in negative-sense RNA at 8 hpi (SND1 KO vs. CTRL). Color scale reflects localization of peak region relative to the 5′ and 3′ end of the viral RNA genome. Peaks with the statistically most pronounced NSP9 binding change are annotated. Initiation site-proximal peaks are encircled. (E) As in (D), but for positive-sense RNA.

As a control, we mapped binding sites of N on viral RNA using eCLIP. We did not observe binding of N to the 5′ end of positive-sense RNA (Figure S6A). Similarly, N did not bind negative-sense RNA at the 3′ end of the SARS-CoV-2 genome (Figure S6A), confirming that the observed binding pattern is a feature of NSP9.

Our results show that the 5′ ends of positive and negative-sense SARS-CoV-2 RNA are covalently linked to NSP9 in infected cells—a phenomenon known as RNAylation. The covalent 5′ end linkage of nascent viral RNA to NSP9 is consistent with NMPylated NSP9 mediating priming of viral RNA synthesis. Importantly, covalent binding of NSP9 to the negative RNA strand is incompatible with an exclusive capping function of NSP9.^59^^,^^60^ Hence, in addition to its role in viral RNA capping,^62^^,^^63^ NSP9 likely primes viral RNA synthesis in positive and negative-sense direction.

### Covalent NSP9-RNA linkages occur at replication-transcription initiation sites

A hallmark of CLIP is the possibility to reveal the nucleotide covalently crosslinked to the protein of interest by mapping reverse transcriptase (RT) termination sites.^41^^,^^69^^,^^70^ The covalent linkage of NSP9 to viral RNA terminates RT reactions in an analogous fashion. Indeed, we observed characteristic RT stops in NSP9 cRIP experiments, including strong RT termination near the 5′ ends of positive and negative-sense RNA (Figure 6B). This termination pattern provides direct evidence that recovered RNA fragments were covalently linked to NSP9 at the respective positions. Therefore, we focus our subsequent analysis on NSP9 peaks supported by adjacent covalent NSP9 linkage sites.

To confirm that the RT termination pattern in SARS-CoV-2 RNA is dependent on covalent linkage of a protein, we performed eCLIP with and without UV-crosslinking for N, which is not expected to be covalently linked to RNA. We observed a striking increase in viral RNA compared to cellular RNA that co-purifies with N upon UV-crosslinking (Figure S6B), indicating that crosslinking stabilizes true interactions over background. Within viral RNA, the majority of significantly enriched RT stops (>82%) reproducibly found in N eCLIP experiments were not enriched over background when omitting UV-crosslinking (Figure S6C, inset). Moreover, a strong shift in the enrichment of crosslinked nucleotides relative to background can be observed upon UV-irradiation (Figure S6C).

Consistent with a function of NSP9 in priming RNA synthesis, the dominant covalent NSP9 linkage site in positive-sense RNA mapped to the first nucleotide of the viral genome (Figure 6B). In negative-sense RNA, we observed the strongest covalent NSP9 linkage at the beginning of the poly(A) tail (Figures 6B and 6C, right). Notably, nucleotides covalently linked to NSP9 in both positive and negative-sense RNA overlap the positions where viral RNA synthesis initiates.^2^ Hence, the pattern of covalent NSP9 linkage in positive and negative-sense RNA is consistent with NSP9 mediating priming of viral RNA synthesis.

We also noticed a covalent NSP9 linkage in negative-sense RNA at nucleotide 76 of the 5′ leader, adjacent to the TRS-leader (TRS-L) sequence (Figure 6C, left). This suggests that NSP9 may prime anti-leader synthesis at this position, which may influence discontinuous transcription or template switching and deserves to be addressed in follow-up studies.

### SND1 depletion leads to imbalanced NSP9 occupancy at initiation sites

Preferential binding of SND1 to negative-sense RNA prompted us to investigate if SND1 impacts binding of NSP9 to RNA. We performed NSP9 cRIP in SND1 KO and control cells infected with SARS-CoV-2 at 8 and 12 hpi. First, we identified peaks significantly enriched relative to SMI in positive and negative-sense RNA and intersected overlapping peaks for each time point. To identify regions with a significant change in NSP9 occupancy, we compared the number of reads in a given peak to the number of reads originating from the remainder of the viral genome (Table S6). At 12 hpi, SND1 KO cells exhibit a loss of NSP9 occupancy at the 5′ end of negative-sense RNA where SND1 binds and RNA synthesis is initiated (Figure 6D). Due to limited abundance and read coverage of negative-sense RNA at 8 hpi, we could not determine differences in NSP9 binding at this time point (Figure S6D). Conversely, at the 5′ end of positive-sense RNA, we observed a significant increase in NSP9 occupancy at 8 and 12 hpi (Figures 6E and S6E). Thus, the 5′ ends of positive and negative-sense RNA are differentially affected by SND1 depletion. In the absence of SND1, NSP9 is redistributed, leading to reduced occupancy at the initiation site for negative-strand RNA synthesis near the 3′ end of the genome, while NSP9 occupancy increases at the 5′ end of the genome.

### SND1 depletion alters the covalent linkage of initiating nucleotides to NSP9

Next, we wondered if NSP9 redistribution upon SND1 depletion affects the function of NSP9 in priming viral RNA synthesis. The covalent 5′ end linkage of viral RNA to NSP9 can be viewed as a characteristic signature of NSP9-mediated priming of SARS-CoV-2 RNA synthesis. Hence, we leveraged the RT termination signature in NSP9 cRIP experiments to quantify changes in the covalent NSP9-RNA linkage upon SND1 depletion (Table S6). We plotted all covalent NSP9 linkages associated with NSP9 peaks in positive and negative-sense RNA across the SARS-CoV-2 RNA genome that exhibit a significant change upon SND1 depletion (Figure 6F). In positive-sense RNA, the first nucleotide of the genome was the strongest differential covalent linkage site. Consistent with our occupancy analysis, the covalent linkage of NSP9 increased at this position upon SND1 depletion. In negative-sense RNA, we observed a significant reduction in covalent NSP9 linkage upon SND1 depletion at nucleotide 29,873, which maps to the beginning of the poly(A) tail (Figure 6F). Hence, differential covalent NSP9 linkages occur at the nucleotides that initiate viral RNA synthesis, suggesting that SND1 depletion impacts priming of viral RNA synthesis by NSP9.

Together, our data suggest a model in which SND1 interacts directly with NSP9 and stimulates recruitment of NSP9 to negative-sense RNA at the 3′ end of the viral genome, thereby improving NSP9-mediated priming of RNA synthesis. In the absence of SND1 the occupancy of NSP9 at initiation sites is imbalanced, which leads to differential NSP9-mediated priming and likely contributes to the SND1 loss-of-function phenotype during SARS-CoV-2 infection.

## Discussion

In this study, we identify SND1 as a host factor that binds negative-sense SARS-CoV-2 RNA and orchestrates nascent viral RNA synthesis through recruitment of NSP9, which likely serves as a protein primer for RNA production.

Selective binding of SND1 to negative-sense SARS-CoV-2 RNA is unusual for several reasons. First, the abundance of negative-sense RNA is at least two orders of magnitude below positive-sense RNA,^17^ suggesting specific recognition by SND1. Second, unlike positive-sense RNA, negative-sense RNA is thought to largely exist in a double-stranded conformation.^64^ Prior work established that SND1 binds dsRNA in human cells.^33^^,^^71^ Intriguingly, the 5′ and 3′ ends are predicted to be the most double-stranded regions in the SARS-CoV-2 genome.^72^ Hence, it is possible that synthesis of negative-sense RNA at the 3′ end of the genome leads to an accumulation of dsRNA, which recruits SND1 and may explain the directionality observed in SND1 binding.

How SARS-CoV-2 initiates RNA synthesis remains poorly understood, and several alternative models exist.^2^^,^^4^^,^^59^ The SARS-CoV-2 RdRP possesses two active sites, one within the NiRAN domain, a characteristic feature of all nidovirus RdRPs.^73^ With the discovery of NSP9 as the target of the NMPylation activity of the NiRAN domain,^59^^,^^60^ a possible role of NSP9 in priming or capping of SARS-CoV-2 RNA came into focus. The 5′ end of coronavirus negative-sense RNA contains a poly(U) tract,^74^ which is compatible with negative-sense RNA synthesis being initiated by base-pairing of UMPylated NSP9 with the poly(A) tail, possibly aided by the RNA-binding activity of NSP9 or its interaction with other RBPs, such as SND1. Consistent with this model, NiRAN-mediated NSP9 NMPylation displays a relaxed nucleotide preference that favors UMP incorporation *in vitro*.^59^^,^^60^ Interestingly, capping of SARS-CoV-2 RNA involves formation of a covalent NSP9-RNA product *in vitro*.^62^^,^^63^ Prior to this study, a covalent NSP9-RNA linkage was only observed with recombinantly purified components *in vitro*.^62^^,^^63^ Our data demonstrate that SARS-CoV-2 RNA is covalently linked to NSP9 via its 5′ end in infected human cells. This is reminiscent of the poliovirus protein VPg, which is uridylated by the RNA polymerase and primes RNA transcription leading to the covalent linkage of the viral genome to VPg.^75^ Surprisingly, we find that in addition to SARS-CoV-2 positive-sense RNA, negative-sense RNA is covalently linked to NSP9 via its 5′ end as well. The proposed role of NSP9 in capping of SARS-CoV-2 RNA^62^^,^^63^ cannot explain covalent binding of NSP9 to negative-sense RNA. Our observations suggest that in addition to its role in capping, NSP9 initiates viral RNA synthesis in positive and negative-sense orientation. Both mechanisms are compatible with each other and it is possible that NSP9 first initiates RNA synthesis before it mediates capping of positive-sense RNA.^60^^,^^65^ This would provide an elegant way to resolve covalent NSP9-RNA products formed during initiation by replacing NSP9 with a 5′ cap.^65^ Finally, the role of NSP9 in priming viral RNA synthesis is compatible with evidence from reverse-genetics studies that suggest a functional interaction between NSP9 and a *cis*-acting RNA element near the 3′ end of the coronavirus genome thought to regulate negative-sense RNA production.^76^ While earlier work report a genetic interaction, we show that SND1 physically interacts with both NSP9 and negative-sense RNA in a region overlapping the previously reported *cis*-acting element.^76^

Remarkably, SND1 modulates the relative occupancy and covalent linkage of NSP9 to initiating nucleotides at the 5′ end of positive and negative-sense viral RNA. Our data are compatible with a model where accumulation of SND1 on negative-sense RNA (Figure 3A) increases the local concentration of its binding partner NSP9 near the site where NMPylated NSP9 initiates RNA synthesis (Figures 6B and 6C). Loss of SND1 impairs this mechanism and results in imbalanced priming of viral RNA synthesis, as evidenced by the differential covalent linkage of NSP9 to the 5′ ends of positive and negative-sense RNA (Figure 6F).

Finally, a genome-wide association study (GWAS) reported rare sequence variants in introns of *SND1* that are associated with severe COVID-19 and increased hospitalization with nominal significance, albeit not at genome-wide significance levels.^77^ This supports the possibility that SND1 is clinically relevant. Our work lays the foundation for better understanding SARS-CoV-2 infections at the molecular level, which opens the door for therapeutic exploitation in follow-up studies.

### Limitations of the study

The work described here expands the known repertoire of RBPs interacting with SARS-CoV-2 RNA at subgenome resolution. While we were able to directly capture positive-sense gRNA and sgmRNA with RAP-MS, the low abundance of negative-sense RNAs prevented their direct capture. Nevertheless, we identified SND1, a protein that preferentially binds negative-sense RNA by mapping its direct RNA contact sites. However, a comprehensive picture of factors that directly bind negative-sense RNA is missing. Additionally, RAP-MS is incompatible with mapping interactions for defined sequence elements within authentic viral RNA, such as the 5′- and 3′-terminal regions of the SARS-CoV-2 genome, which will be a desirable goal once suitable techniques are available. Future work will also need to illuminate the exact molecular feature(s) driving SND1 binding to negative-sense RNA of SARS-CoV-2 or other coronaviruses.

## STAR★Methods

### Key resources table

**Table:** 

REAGENT or RESOURCE	SOURCE	IDENTIFIER
**Antibodies**		

Anti-SND1 antibody (rabbit)	Bethyl	Cat# A302-883A, RRID:AB_10631268
SARS-CoV-2 (COVID-19) NSP9 antibody (rabbit)	GeneTex	Cat# GTX135732, RRID:AB_2909871
SARS-CoV-2 (COVID-19) nucleocapsid antibody (rabbit)	GeneTex	Cat# GTX135357, RRID:AB_2868464
SARS-CoV-2 (COVID-19) NSP3 antibody (rabbit)	GeneTex	Cat# GTX135614, RRID:AB_2887505
Normal Rabbit IgG	Cell Signaling Technology	Cat# 2729, RRID:AB_1031062
Anti-SND1 antibody (mouse)	Proteintech	Cat# 60265-1-Ig, RRID:AB_2881386
Anti-SND1 antibody (rabbit)	Proteintech	Cat# 10760-1-AP, RRID:AB_2193410
Anti-dsRNA J2 antibody (mouse)	Jena Bioscience	RNT-SCI-10010200
Anti-FLAG (mouse)	Sigma-Aldrich	Cat# F3165, RRID:AB_259529
Anti-V5 (rabbit)	Abcam	Cat# ab9116, RRID:AB_307024
Anti-Actin (rabbit)	Sigma Aldrich	Cat# A2103, RRID:AB_476694

**Bacterial and virus strains**		

SARS-CoV-2	Peterhoff et al.^78^	N/A

**Chemicals, peptides, and recombinant proteins**		

Dynabeads Protein G	Thermo Fisher Scientific	10004D
T4 Polynucleotide Kinase	New England Biolabs	M0201L
FastAP Thermosensitive Alkaline Phosphatase	Thermo Fisher Scientific	EF0651
100% DMSO	Sigma-Aldrich	41639
50% PEG8000	New England Biolabs	B10045
T4 RNA ligase high concentration	New England Biolabs	M0437M
5 M NaCl	VWR International	A7006.1000
Proteinase K	New England Biolabs	P8107S
0.1 M DTT	Thermo Fisher Scientific	Y00147
Lithium Chloride	Merck Millipore	5922-500ML
Dodecyl maltoside	Carl Roth	CN26.5
Sodium dodecyl sulphate	Omnilab	APP A3942.1000
Sodium deoxycholate	Sigma-Aldrich	30970-25G
IGEPAL CA-630	Sigma-Aldrich	I8896-100ML
Complete EDTA-free protease inhibitor cocktail	Sigma Aldrich	11873580001
Murine RNase inhibitor	New England Biolabs	M0314L
Turbo DNase	Thermo Fisher Scientific	AM2239
EDTA	VWR International	A4892.1000
EGTA	Carl Roth	3054.1
Urea	Sigma-Aldrich	U0631
TCEP	Sigma-Aldrich	646547
16% Formaldehyde (w/v), Methanol-free	Thermo Fisher Scientific	28908
4',6-Diamidino-2-Phenylindole (DAPI)	Thermo Fisher Scientific	D1306
Fluoromount-G Mounting Medium	Thermo Fisher Scientific	00495802
Dynabeads MyOne Streptavidin C1	Thermo Fisher Scientific	65002
Dynabeads MyOne Silane	Thermo Fisher Scientific	37002D
AMPure XP beads	Beckmann Coulter	A63881
NHS-activated magnetic beads	Thermo Fisher Scientific	PIER88827
N-laroylsarcosine	VWR International	SAFSL7414-50ML
Benzonase	Merck Millipore	71206-3
RNase I	Thermo Fisher Scientific	AM2295
ExoSAP-IT	Thermo Fisher Scientific	78201.1.ML
SUPERase-In RNase Inhibitor	Thermo Fisher Scientific	AM2696
Trichloroacetic acid	Sigma-Aldrich	T0699-100ML
Lipofectamine 2000 transfection reagent	Thermo Fisher Scientific	11668019
FuGene HD transfection reagent	Promega	E2311
NuPAGE 4-12% Bis-Tris Protein Gels	Thermo Fisher Scientific	NP0335BOX
NuPAGE 3-8% Tris-Acetate Protein Gels	Thermo Fisher Scientific	EA0378BOX
AffinityScript reverse transcriptase	Agilent	600109
PowerUp SybrGreen master mix	Thermo Fisher Scientific	A25778
SsoAdvanced Universal SYBR Green Supermix	Biorad Laboratories	1725272
4-Thiouridine	Biosynth Carobsynth	NT06186
Iodoacetamide	Thermo Fisher Scientific	A39271
Buffer RLT	Qiagen	79261
Poly-A-Polymerase	Takara	2180A
ATP	New England Biolabs	N0437A
SMARTscribe Reverse Transcriptase	Takara	639538
Q5 Hot Start High-Fidelity 2x Master Mix	New England Biolabs	M0494L
Deoxynucleotide Mix	Agilent Technologies	200415
Lipofectamine RNAiMAX	Life Technologies	13778030
Anti-FLAG® M2 Magnetic Beads	Merck Millipore	M8823
Proteintech ChromoTek V5-Trap Magnetic Agarose	Proteintech	V5TMA
Triton-X 100	Sigma-Aldrich	T9284-100ML
RNase H	New England Biolabs	M0297L
4x LDS sample buffer	Thermo Fisher Scientific	B0007
QX200 ddPCR EvaGreen Supermix	BioRad	1864034
QX200 droplet generation oil for EvaGreen	BioRad	1864006
2′-deoxythymidine-3′,5′-bisphosphate	Biosynth	NT09781
SND1 recombinant protein	Origene	TP300059
NSP9 recombinant protein	Abcam	ab289608

**Critical commercial assays**		

Pierce BCA Protein Assay Kit	Thermo Scientific	Cat# 23225
CellTiter-Glo® Luminescent Cell Viability Assay	Promega	G7571
Duolink in situ red starter kit	Sigma Aldrich	DUO92101-1KT
Duolink in situ detection reagents green	Sigma Aldrich	DUO92014-30RXN
HCR IHC + HCR RNA-FISH Kit	Molecular Instruments	N/A
Qubit dsDNA HS Kit	Thermo Fisher Scientific	Q32851
Direct-zol RNA MicroPrep	Zymo Research	R2063
RNA Clean & Concentrator-5	Zymo Research	R1015
Zymoclean Gel DNA Recovery Kit	Zymo Research	D4007
NEB Next rRNA Depletion Kit v2	New England Biolabs	E7400X
RED-MALEIMIDE 2nd Generation kit	NanoTemper Technologies	SKU: MO-L014

**Deposited data**		

Illumina sequencing data	This paper	GEO: GSE217429
CoIP proteomics data	This paper	PRIDE: PXD037528
RAP-MS proteomics data	This paper	MassIVE: MSV000090682
Source code for quantification and statistical analysis	This paper	https://github.com/AlexGa/cRIP-eCLIP-workflow and https://doi.org/10.5281/zenodo.8304669

**Experimental models: Cell lines**		

Human: Huh-7 cell line	Virology Diagnostics Unit at Institute of Virology and Immunobiology, University of Würzburg	N/A
Human: Calu-3 cell line	Virology Diagnostics Unit at Institute of Virology and Immunobiology, University of Würzburg	N/A
Human: A549^ACE2^ cell line	A. Pichlmair	N/A
African Green Monkey: Vero-E6-TMPRSS2 cell line	S. Pöhlmann	N/A
Human: HEK293T	U. Fischer	N/A

**Oligonucleotides**		

RiL-19: /5Phos/rArGrArUrCrGrGrArArGrArGrCrArCrArCr GrUrC/3SpC3/	van Nostrand et al.^41^	N/A
AR17: CAGACGTGTGCTCTTCCGA	van Nostrand et al.^41^	N/A
Rand103Tr: /5Phos/NNNNNNNNNNAGATCGGAAGA GCGTCGTGT/3SpC3/	van Nostrand et al.^41^	N/A
Universal forward PCR primer: AATGATACGGCGACCACCGAGATCTACACTCTTTC CCTACACGACGCTCTTCCGATCT	van Nostrand et al.^41^; Illumina customer service letter	N/A
Barcoded reverse PCR primer: CAAGCAGAAGACGG CATACGAGATNNNNNNNNGTGACTGGAGTTCAGA CGTGTGCTCTTCCGATCT	van Nostrand et al.^41^; Illumina customer service letter	N/A
Biotinylated antisense probes	Schmidt et al.^8^	N/A
HCR probes NegativeSense_N	Molecular Instruments	PRK126
ON-TARGETplus Human SND1 (27044) siRNA - SMARTpool	Dharmacon	L-010657-01-0005
ON-TARGETplus Human ACE2 (59272) siRNA - SMARTpool	Dharmacon	L-005755-00-0005
ON-TARGETplus Non-targeting Pool	Dharmacon	D-001810-10-20
Template-switching oligo: Biotin/TACACGACGCTCTT CCGATCTrGrG + G	Basak et al.^79^	N/A
RT primer: CAGACGGCTCTTCCGATCTTTTTTTTTTTT TTTTTTTTTTTTTTTTTTTV-3′	This paper	N/A
RdRP Fwd qPCR primer: GTGARATGGTCATGTGTGGCGG	Corman et al.^80^	N/A
RdRP Rev qPCR primer: CARATGTTAAASACACTATTAGCATA	Corman et al.^80^	N/A
N Fwd qPCR primer: CACATTGGCACCCGCAATC	Corman et al.^80^	N/A
N Rev qPCR primer: GAGGAACGAGAAGAGGCTTG	Corman et al.^80^	N/A
18S rRNA Fwd qPCR primer: ATGGCCGTTCTTAGTTGGTG	Schmidt et al.^8^	N/A
18S rRNA Rev qPCR primer: GAACGCCACTTGTCCCTCTA	Schmidt et al.^8^	N/A
SARS-CoV-2 leader Fwd qPCR primer: CCCAGGTAACAAACCAACCAAC	This paper	N/A
SARS-CoV-2 ORF1a (gRNA) Rev qPCR primer: CTCGTTGAAACCAGGGACAAG	This paper	N/A
SARS-CoV-2 M (sgmRNA) Rev qPCR primer: GGTTCCATTGTTCAAGGAGCTT	This paper	N/A
SARS-CoV-2 N (sgmRNA) Rev qPCR primer: GTAATGCGGGGTGCATTTCG	This paper	N/A

**Recombinant DNA**		

pLEX_307	David Root	Addgene plasmid #4139
SND1 (NM_014390) Human Tagged ORF Clone	Origene	RC200059
pDONR223 SARS-CoV-2 NSP9	Fritz Roth	Addgene plasmid #141263
pLEX-SND1	This paper	N/A
pLEX-SND1-FLAG	This paper	N/A
pLEX-NSP9-V5	This paper	N/A
CRISPR/Cas9 control plasmid	Santa Cruz Biotechnology	sc-418922
SND1 CRISPR/Cas9 KO plasmids	Santa Cruz Biotechnology	sc-403949

**Software and algorithms**		

Spectrum Mill MS Proteomics Software	Broad Institute	https://proteomics.broadinstitute.org/millhome.htm
ImageJ	Schneider et al.^81^	https://imagej.nih.gov/ij/
R	The Comprehensive R Archive Network	https://cran.r-project.org/
Python	Python Programming Language	https://www.python.org/
BWA	Li and Durbin^82^	http://bio-bwa.sourceforge.net/bwa.shtml
Picard Tools	Broad Institute	https://broadinstitute.github.io/picard
Samtools	Li et al.^83^	http://samtools.sourceforge.net/
Bedtools	Quinlan and Hall^84^	https://github.com/arq5x/bedtools2/blob/master/docs/content/overview.rst
MACS2	Zhang et al.^85^	https://github.com/macs3-project/MACS
Trimmomatic	Bolger et al.^86^	http://www.usadellab.org/cms/index.php?page=trimmomatic
STAR	Dobin et al.^87^	https://github.com/alexdobin/STAR
IGV	Robinson et al.^88^	https://software.broadinstitute.org/software/igv/
Big-FISH	Imbert et al.^89^	https://big-fish.readthedocs.io/en/stable/
DAVID Bioinformatics Resources	https://david.ncifcrf.gov/	DAVID 6.8

### Resource availability

#### Lead contact

Further information and requests for resources and reagents should be directed to and will be fulfilled by the lead contact, Mathias Munschauer (mathias.munschauer@helmholtz-hiri.de).

#### Materials availability

Cell lines and plasmids generated in this study are available upon request from the lead contact.

### Experimental model and study participant details

#### Cell culture

We maintained Huh-7 (human), A549^ACE2^ (human), HEK293T (human), Calu-3 (human), and Vero-E6-TMPRSS2 (African green monkey) cells in DMEM (Thermo Fisher Scientific) supplemented with 10% heat-inactivated FBS (Thermo Fisher Scientific), and 100 units/ml streptomycin and 100 mg/ml penicillin. Cells were grown at 37°C and 5% CO_2_ atmosphere. A549^ACE2^ cells were a gift from A. Pichlmair and Vero-E6-TMPRSS2 cells were a gift from S. Pöhlmann.

#### Virus stock production

A SARS-CoV-2 patient isolate^78^ was passaged up to three times in Vero-E6-TMPRSS2 cells. For generation of virus stocks, Vero-E6-TMPRSS2 cells were infected at MOI 0.01 PFU/cell. Cells were incubated with the inoculum for 2 h at 37°C, the inoculum was removed and OptiMem (Gibco) containing 1% FBS was added. At 48-72 h post-infection, the supernatant was cleared by centrifugation (500g, 10 min, 4°C), aliquoted and stored at -80°C. Viral titers were determined by plaque assay on Vero-E6-TMPRSS2 cells as described previously.^8^ All infection experiments were conducted using viral titers determined on Vero-E6-TMPRSS2 cells. Titers determined on A549^ACE2^ cells using identical virus stocks were substantially reduced (Figure S3C).

### Method details

#### RNA antisense purification and mass spectrometry (RAP-MS)

##### Infection, UV-crosslinking and cell lysis

For RAP-MS experiments, around 480 million A549^ACE2^ or Huh-7 cells were infected with SARS-CoV-2 at MOI 5 PFU/cell. At 24 h post-infection, cells were washed with cold PBS and irradiated with 254 nm UV light on ice at a total dose of 800 mJ/cm^2^ in a GS Gene Linker (Bio-Rad Laboratories). Cells were lysed in 1 ml RAP lysis buffer (10 mM Tris pH 7.5, 500 mM LiCl, 0.5% dodecyl maltoside (DDM), 0.5% sodium dodecyl sulphate (SDS), 0.1% sodium deoxycholate, EDTA-free Protease Inhibitor Cocktail (Sigma Aldrich, 11873580001) and 300 U/ml Murine RNase Inhibitor (NEB, M0314L)) per 10 million cells, collected by scraping, and lysates were stored at -80°C.

##### Lysate preparation

Lysates were homogenized by pipetting and passing through a needle (1.2 mm and 0.8 mm diameter) followed by sonication on ice (Branson sonicator) at 10% power at 0.7s on, 2.2s off with a total energy input of ∼2-3 kJ. DNA was digested by adding 30 U/ml TURBO DNase (Thermo Fisher Scientific, AM2239), 2.5 mM MgCl_2_, and 0.5 mM CaCl_2_ and incubated 20 min at 37°C. DNase was quenched by adding 10 mM EDTA, 5 mM EGTA, and 2.5 mM TCEP. We added 2 volumes of 6 M Urea buffer (10 mM Tris pH 7.4, 5 mM EDTA, 500 mM LiCl, 0.5% DDM, 0.05% SDS, 0.1% sodium deoxycholate, 6 M Urea, 2.5 mM TCEP) and incubated the lysates 15 min on ice, followed by centrifugation at 5000 g for 15 min at 4°C to remove any residual cell debris. To pre-clear the lysates, 1 ml MyOne Streptavidin C1 Dynabeads (Thermo Fisher Scientific, 65002) per 100 million cells were washed 4 times with 10 mM Tris pH 7.4 followed by 2 washes with hybridization buffer (10 mM Tris pH 7.4, 5 mM EDTA, 500 mM LiCl, 0.5% DDM, 0.2% SDS, 0.1% sodium deoxycholate, 4 M Urea, 2.5 mM TCEP) and added to the cell lysate. Lysates and beads were incubated at 37°C for 30 min while shaking at 800 rpm and beads were removed from the lysates by magnetic separation.

##### RNA antisense purification

We designed and synthesized 5′ biotinylated 90-mer DNA oligonucleotides (Integrated DNA Technologies) antisense to the complementary target RNA sequence. We used 67 probes such that one probe binding site occurs roughly every 400 bases in the ∼30 kb SARS-CoV-2 genome and excluded regions that matched to human transcripts or genomic regions as previously described.^8^ Lysates were divided into 3 equal parts and antisense purification was performed using the full antisense probe set for SARS-CoV-2 RNA, or a subset of probes covering ORF1ab (nucleotides 1-21509 of the SARS-CoV-2 genome) to capture SARS-CoV-2 gRNA, or RMRP antisense probes as control. We denatured the probes at 85°C for 3 min, added 20 μg of biotinylated antisense probe per 160 million cells and incubated probes and lysates for 2 h at 55°C at 800 rpm. Per 160 million cells, 2 ml streptavidin beads were washed as described above and added to the lysates. Lysates and beads were incubated at 55°C for 30 min, the flow-through was removed and the beads washed 4 times with 2 ml hybridization buffer at 55°C. The flow-through of the gRNA purification sample was incubated with fresh MyOne Streptavidin C1 Dynabeads to remove any residual capture probes by following the pre-clear procedure described above. Following a 30 min incubation, pre-clear beads were discarded and the flow-through was subjected to a second round of antisense purification using a set of antisense probes binding to nucleotides 21600-29789 of the SARS-CoV-2 genome to capture sgmRNAs by following procedures described above.

##### Protein elution

To elute proteins, beads were rinsed with benzonase elution buffer (20 mM Tris pH 8.5, 2 mM MgCl_2_, 0.05% N-laroylsarcosine (NLS), 0.5 mM TCEP) while magnetically separated and incubated with 500 U benzonase (Merck Millipore, 71206-3) and 44 U RNase I (Thermo Fisher Scientific, AM2239) in benzonase elution buffer at 37°C and 1000 rpm overnight. The supernatant was separated from the beads and transferred into new tubes. To precipitate proteins, 100% Trichloroacetic acid (TCA) was added to a final concentration of 20%, followed by incubation on ice for 2 h and centrifugation at 14,000 g, 4°C for 1 h. The pellet washed once in ice-cold acetone, air-dried and dissolved in 8 M urea in 50 mM Tris pH 8.5.

##### Protein digestion and TMT labeling

Two replicates of each RNA purification (all viral RNA, gRNA, sgmRNA, and RMRP) were jointly analyzed in a single TMT cassette. Reconstituted protein pellets were reduced with 4 mM dithiothreitol (DTT) for 30 min at room temperature, followed by alkylation with 10 mM iodoacetamide (IAA) for 45 min at room temperature in the dark. Samples were then digested with 0.1 μg Lys-C for 2 h, followed by a reduction of the urea concentration to <2 M and continued digestion with 0.5 μg trypsin overnight. Reactions were quenched with formic acid at a final concentration of 5% and then desalted by reverse phase C18 stage tips as described previously^90^ and dried down. Peptides were then resuspended in 50 μl of 50 mM HEPES buffer and isobarically labeled using 400 μg of eight of the channels of TMT10-plex isobaric labeling reagent (Thermo Fisher Scientific). The labeling reactions were then quenched with 4 μl of 5% hydroxylamine, samples were mixed together, and dried. The sample was fractionated by SCX stage tip strategy using three pH cuts at 5.15, 8.25, and 10.3 as described previously.^90^

##### LC-MS/MS analysis

Samples were analyzed on an Orbitrap Exploris 480 mass spectrometer coupled with Easy-nLC 1200 ultra-high pressure liquid chromatography (UPLC) system (Thermo Fisher Scientific) with solvent A of 0.1% formic acid (FA)/3% acetonitrile and solvent B of 0.1% FA/90% acetonitrile. Half of each of the RAP-MS fraction were injected on a 75 μm ID Picofrit column packed in-house to approximately 28 cm length with Reprosil-Pur C18-AQ 1.9 μm beads (Dr. Maisch GmbH). Samples were separated at 200 nL/min flow rate with a gradient of 2-6% solvent B for 1 min, 6-30% B in 84 min, 30-60% B in 9 min, 60-90% B in 1 min, followed by a hold at 90% B for 5 min. The mass spectrometer was operated in data-dependent acquisition mode. On Exploris 480 MS1 scan (r = 60,000) was followed by MS2 scans (r = 45,000) for top 20 most abundant ions using normalized automatic gain control (AGC) of 100% for MS1 and 200% for MS2, MS2 maximum inject time of 150 ms, normalized collision energy of 34 and fit filter of 50%.

#### Generation of SND1 knockout cell lines

A549^ACE2^ or Huh-7 cells were seeded in a 6-well plate (2.5x10^5^ cells/well) and transfected the next day with 2.5 μg SND1 CRISPR/Cas9 KO plasmids (Santa Cruz Biotechnology, sc-403949) or a CRISPR/Cas9 control plasmid (Santa Cruz Biotechnology, sc-418922), using 2 μl Lipofectamine 2000 transfection reagent (Thermo Fisher Scientific) per 1 μg DNA. A plasmid containing a puromycin resistance gene was co-transfected as selection marker and successfully transfected cells were selected with puromycin (5 μg/ml) starting at 24 h post-transfection for 2 days. Clonal cell lines were obtained from the surviving cell population by limited dilution and SND1 expression was analyzed by western blot.

#### Plasmids

Plasmids for SND1 complementation were generated using C-terminally Myc-DDK-tagged-Human SND1 (NM_014390), which was purchased from Origene and subcloned into pDONR221 removing the C-terminal Myc-DDK tag. Untagged SND1 was then cloned into pLEX_307 (a gift from David Root, Addgene #41392) using Gateway cloning. To generate a pLEX-empty control vector, gateway attB1/2 sites were removed from pLEX by digesting with BamHI and EcoRV. To generate SND1 domain deletion constructs, a single C-terminal Flag tag followed by a stop codon was fused to SND1 in the pDONR221 backbone. Sequence regions corresponding to individual protein domains (SN1: 26-166, SN2: 193-328, SN3: 341-496, SN4: 525-660, Tudor: 729-787, SN5: 844-895) were removed by site-directed mutagenesis and resulting constructs were cloned into pLEX_307 using Gateway cloning. Untagged pDONR223 SARS-CoV-2 NSP9 (a gift from Fritz Roth, Addgene #141263) was cloned into pLEX_307 using Gateway cloning.

#### Lentivirus production and transduction

To produce lentiviral particles, HEK293T cells were transfected with psPax, MD2-G and plex lentiviral vector at a ratio of 3:2:5 using FuGene HD transfection reagent (Promega, E2311) (3 μl transfection reagent per 1 μg plasmid DNA). Lentivirus-containing supernatants were harvested at 48 h post-transfection, cleared by centrifugation (500 g, 5 min, 4°C) and filtered through a 0.45 μm filter. A549^ACE2^ SND1 knockout cells or Huh-7 SND1 knockout cells were transduced with lentiviruses to reconstitute SND1 expression, or empty control vector and subjected to puromycin selection (1 μg/ml) at 48 h post-transduction.

#### Virus infections

In general, we seeded 1x10^5^ cells per well of a 24-well plate and infected them with SARS-CoV-2 the next day. Virus inoculum was prepared in DMEM containing 5% FBS and 100 units/ml streptomycin and 100 mg/ml penicillin. Cells were washed with pre-warmed PBS and incubated with the inoculum for 1 h at 37°C. The inoculum was removed, cells were washed with PBS, and fresh DMEM supplemented with 5% FBS and 100 units/ml streptomycin and 100 mg/ml penicillin was added to the cells. Infection of A549^ACE2^ cells at MOI = 3 PFU/cell did not lead to any detectable cytopathic effect. Where indicated, cells were pre-treated with 2′-deoxythymidine-3′,5′-bisphosphate (pdTp) (Biosynth, NT09781) for 21 h before infection with SARS-CoV-2 and pdTp was added to the medium after infection. Cell viability assay was performed in uninfected cells at 27 h after addition of pdTp using the CellTiter-Glo reagent (Promega, G7571) according to the manufacturer’s instructions.

#### SARS-CoV-2-GFP reporter infections

A549^ACE2^ SND1 knockout or control cell lines were infected with SARS-CoV-2-GFP^40^ at an MOI of 3. The infection was monitored with an IncuCyte S3 live-cell fluorescent microscope (Sartorius) by acquiring the GFP signal and phase-contrast images every 4 h for a total time of 36 h. The GFP positive area was then normalized to the confluence per image, after Top-Hat background removal (radius: 30 μm, threshold: 0.6 green-calibrated units (GCU), edge-split: off) using the built-in IncuCyte analysis software (2020C Rev1).

#### Western blot

For western blot analysis, 2x10^5^ cells were rinsed with PBS and lysed in 80 μl Laemmli buffer (2% SDS, 10% Glycerol, 60 mM Tris, 0.01% (w/v) bromphenol blue, 50 mM DTT) and lysates were sheared by passing through a syringe. Proteins were separated by SDS PAGE in NuPAGE 4-12% Bis-Tris Protein Gels (Thermo Fisher Scientific) or 3-8% Tris-Acetate Protein Gels (Thermo Fisher Scientific) and transferred to a nitrocellulose membrane using the iBlot dry blotting system (Thermo Fisher Scientific). Membranes were incubated with anti-SND1 (Proteintech, 60265-1-Ig), anti-Flag (Sigma, F3165), anti-HA (Proteintech, 66006-2-Ig), anti-V5 (Abcam, ab9116) or anti-Actin (Sigma Aldrich, A2103) antibodies using the iBind Automated Western System (Thermo Fisher Scientific) and imaged in the Odyssey Clx Infrared Imager System (LI-COR).

#### Co-immunoprecipitation and western blot

HEK293T cells were plated in a T25 flask at a density of 1.7 x10^6^ cells per flask and the next day transfected with 1.35 μg pLEX-NSP9-V5, 1.35 μg pLEX-SND1-Flag expression plasmids (containing wildtype SND1 or SND1 domain deletion constructs), and FuGene HD transfection reagent (Promega, E2311) (3 μl transfection reagent per 1 μg plasmid DNA). At 24 hours post-transfection, cells were washed once with ice-cold PBS and lysed in 200 μl Flag lysis buffer (50 mM Tris-HCl pH 7.5, 150 mM NaCl, 1% (v/v) Triton-X 100, complete EDTA-free protease inhibitor cocktail) for 30 min on ice. Lysates were cleared by centrifugation at 14 000 *g* and 4°C for 10 minutes. Where indicated, Benozonase nuclease (Merck Millipore, 71206) was added to the lysates to a final concentration of 0.25 U/μl, followed by an incubation for 10 min at room temperature. Lysates were incubated with 50 μl Anti-Flag M2 magnetic beads (Merck Millipore, M8823) for 2h at 4°C with gentle rotation. Next, beads were washed 4 times with Flag IP wash buffer (50 mM Tris pH 7.5, 150 mM NaCl, 0.1 % TritonX-100) and proteins were eluted from the beads by adding 40 μl 2x Laemmli buffer (120 mM Tris-HCl pH 6.8, 10% SDS, 20% Glycerol, bromophenol blue) and analyzed by western blot. For V5 IPs, we applied the following modifications to the described procedure: cells were lysed in V5 lysis buffer (10 mM Tris-HCl pH 7.5, 150 mM NaCl, 0.5 mM EDTA, 0.5% (v/v) IGEPAL, complete EDTA-free protease inhibitor cocktail), and after clearing of the cell lysates, 1.5x volumes dilution buffer (10 mM Tris-HCl pH 7.5, 150 mM NaCl, 0.5 mM EDTA, complete EDTA-free protease inhibitor cocktail) were added. The lysates were incubated with 25 μl ChromoTek V5-Trap magnetic agarose beads (chromotek, V5TMA) and beads were washed 4 times with V5 wash buffer (10 mM Tris pH 7.5, 150 mM NaCl, 0.5 mM EDTA, 0.2 % IGEPAL).

#### RNA extraction and reverse-transcriptase quantitative PCR

Approximately 2x10^5^ cells were lysed in 300 μl Trizol/well and RNA was extracted using the Direct-zol RNA MicroPrep Kit (Zymo Research, R2063). RNA was reverse transcribed into cDNA using Affinity Script reverse transcriptase (Agilent, 600109) according to the manufacturer’s instructions using random primers. Viral RNA was quantified by qPCR using PowerUp SYBR Green master mix (Thermo Fisher Scientific, A25778) or SsoAdvanced Universal SYBR Green Supermix (Biorad Laboratories, 1725272) and specific primers for viral RdRP gene (Fwd: 5ʹ-GTGARATGGTCATGTGTGGCGG, Rev: CARATGTTAAASACACTATTAGCATA-3ʹ), viral N gene (Fwd: 5ʹ-CACATTGGCACCCGCAATC-3ʹ, Rev: 5ʹ-GAGGAACGAGAAGAGGCTTG-3ʹ) or 18S ribosomal RNA (Fwd: 5ʹ-ATGGCCGTTCTTAGTTGGTG-3ʹ, Rev: 5ʹ-GAACGCCACTTGTCCCTCTA-3ʹ). To specifically analyze viral sgmRNAs and gRNA, we designed a common forward primer that binds within the SARS-CoV-2 leader sequence (5ʹ-CCCAGGTAACAAACCAACCAAC-3ʹ) and a reverse primer specific for the ORF1a RNA (5ʹ-CTCGTTGAAACCAGGGACAAG-3ʹ), the mRNA of M (5ʹ-GGTTCCATTGTTCAAGGAGCTT-3ʹ), or the mRNA of N (5ʹ-GTAATGCGGGGTGCATTTCG-3ʹ).

We performed 4 technical qPCR replicates (from the same cDNA) and took the median value for further analysis. To calculate differences in RNA expression we used the ΔΔCT method versus 18S rRNA.

#### Digital droplet PCR

For absolute quantification of viral RNA, we subjected cDNA samples from infected cells to digital droplet PCR (ddPCR) using a common forward primer that binds within the SARS-CoV-2 leader sequence (5ʹ-CCCAGGTAACAAACCAACCAAC-3ʹ) and a reverse primer binding in the ORF1a gene to detect genomic RNA (5ʹ-CTCGTTGAAACCAGGGACAAG-3ʹ), or the N sgmRNA (5ʹ-GTAATGCGGGGTGCATTTCG-3ʹ). We mixed QX200 ddPCR EvaGreen Supermix (BioRad, 1864034) with 200 nM of each primer and cDNA, and generated droplets using the QX200 droplet generator (BioRad) and QX200 droplet generation oil for EvaGreen (BioRad, 1864006) according to the manufacturer’s instructions. After performing the PCR, the signal from individual droplets was detected in a QX200 droplet reader (BioRad) and data was analyzed using the QX Manager Standard Edition 2.0 software (BioRad).

#### siRNA knockdown

For transient siRNA knockdown experiments in a 24-well plate format, a transfection mix containing a final concentration of 50 nM ON-TARGETplus siRNA SMARTpool targeting SND1 (Dharmacon, L-010657-01-0005), ACE2 (Dharmacon, L-005755-00-0005) or a non-targeting control siRNA pool (Dharmacon, D-001810-10-20) and 1.5 μl Lipofectamine RNAiMAX (Life Technologies, 13778030) per well was prepared in OptiMEM. Next, 2.5x10^4^ A549^ACE2^ cells or 9x10^4^ Calu-3 cells were seeded on top of the transfection mix and incubated for 72 hours before infection with SARS-CoV-2. For optimization experiments with shorter incubation periods, 1x10^5^ A549^ACE2^ cells or 1.8 x10^5^ Calu-3 cells were seeded per well for harvesting at 24 hours post-transfection, and 5x10^4^ A549^ACE2^ cells or 1.35x10^5^ Calu-3 cells were seeded per well for harvesting at 48 hours post-transfection

For cell viability assays, 5x10^3^ A549-ACE2 cells or 1.8x10^4^ Calu-3 cells were reverse transfected in a 96-well plate format with a final concentration of 50 nM siRNA and 0.3 ul Lipofectamine RNAiMAX (Life Technologies, 13778030) per well. At 72 hours post-transfection, cell viability was analyzed using the CellTiter-Glo assay (Promega, G7571) according to the manufacturer’s instructions.

#### eCLIP-seq and covalent RIP-seq

##### Infection and cell lysis

For eCLIP and cRIP experiments, we grew 24 million A549^ACE2^ or Huh-7 cells per condition and infected them with SARS-CoV-2 at MOI 3 PFU/cell. At indicated time points, culture media was removed, cells were rinsed with cold PBS and subjected to UV-irradiation with a total dose of 0.8 J/cm^2^ on ice and scraped in cold PBS. For cRIP-seq experiments, cells were scraped in cold PBS without UV-crosslinking. Cells were pelleted by centrifugation (200 g, 8 min, 4°C) and lysed by adding 2x eCLIP lysis buffer (100 mM Tris-HCl pH 7.5, 300 mM NaCl, 2 mM EDTA, 2% (v/v) IGEPAL CA-630, 1% sodium deoxycholate, 0.5 mM TCEP, EDTA-free Protease Inhibitor Cocktail (Sigma Aldrich, 11873580001)) for eCLIP-seq experiments, or 2x Co-IP buffer (100 mM Tris-HCl pH 7.4, 300 mM NaCl, 2% (v/v) IGEPAL CA-630, 1% sodium deoxycholate, 0.5 mM TCEP, EDTA-free Protease Inhibitor Cocktail (Sigma Aldrich, 11873580001)) for cRIP-seq. Following a 30 min incubation at room temperature, lysates were used for immunoprecipitation procedures and sequencing libraries were prepared as outlined below.

##### Immunoprecipitation and library preparation

Fresh lysates were combined with an equal amount of nuclease-free water to adjust the respective lysis buffer to a 1x concentration. Lysates were sonicated and RNase I (Thermo Fisher Scientific, AM2295) was added (1:100 of a 1:25 stock dilution), followed by a 10 min incubation at 22°C to achieve limited RNA digestion. Next, DNA was digested for 20 min at 37°C with Turbo DNase (Thermo Fisher Scientific, AM2239) using a 1:250 dilution and adding 2.5 mM MgCl_2_ and 0.5 mM CaCl_2_. Samples were cooled on ice for 5 min and subsequently centrifuged at 5000 g for 15 min at 4°C. The supernatant was transferred, and its protein concentration was determined using a BCA assay. The resulting lysates were pre-cleared for 45 min at 4°C by incubating them with uncoupled Protein G beads (Thermo Fisher Scientific, 10004D). Five percent of the pre-cleared lysates were used to generate SMI controls (see below).

For every mg of total protein, 5 μg antibody (Anti-SND1 antibody, Bethyl, A302-883A; Anti-Nsp9 antibody, GeneTex, GTX135732; Anti-N antibody, GeneTex, GTX135357) were coupled to 30 μl Protein G beads for 1 h at room temperature. The antibody-coupled beads were added to the pre-cleared lysate for overnight incubation at 4°C on a rotating wheel. After removing supernatants, the beads were washed twice with eCLIP lysis buffer, twice with IP wash buffer (50 mM Tris-HCl pH 7.4, 300 mM NaCl, 1 mM EDTA, 1 % (v/v) IGEPAL CA-630, 0.5 % Sodium Deoxycholate, 0.25 mM TCEP) and twice with IP no salt buffer (50 mM Tris-HCl pH 7.4, 1 mM EDTA, 0.5 % (v/v) IGEPAL CA-630). Beads were then rinsed with 1x Fast alkaline phosphatase (AP) buffer and RNA dephosphorylation was performed by adding FastAP mix (39 μl H_2_O, 5 μl 10x FastAP buffer, 5 μl FastAP enzyme (Thermo Fisher Scientific), 1 μl murine RNase inhibitor) and incubating for 20 min at 37°C while shaking at 800 rpm. To ensure full dephosphorylation, polynucleotide kinase (PNK) mix (120 μl H_2_O, 20 μl 10x PNK buffer, 7 μl T4 PNK enzyme (NEB), 2 μl Murine RNase inhibitor (NEB), 1 μl Turbo DNase (Thermo Fisher Scientific)) was added and incubated for another 20 min at 37°C. Afterwards, ice-cold no salt IP wash buffer was added, and samples were incubated on ice for 5 min. Beads were washed once in cold RNA ligase buffer and RNA ligation mix (9 μl H_2_O, 9 μl 50% PEG 8000, 3 μl 10x RNA ligase buffer (NEB) without DTT, 2.5 μl T4 RNA ligase high concentration (NEB), 0.8 μl DMSO, 0.4 μl Murine RNase inhibitor (NEB), 0.3 μl 0.1 M ATP) was added together with 2.5 μl RiL-19 adapter and 2.5 μl H_2_O and incubated for 1 h 15 min at 23°C with intermittent shaking. 1 ml ice-cold no salt IP wash buffer was added, and sample was incubated on ice for 5 min. The supernatant was removed, and beads were resuspended in 40 μl 1x LDS sample buffer (Thermo Fisher Scientific), containing 12.5 mM TCEP.

Next, 30 μl of the SMI samples were mixed with 10 μl 4x LDS sample buffer, containing 50 mM TCEP and incubated at 80°C for 10 min together with the IP samples. Both IP and SMI samples were separated by SDS-PAGE, transferred on nitrocellulose membranes, and the expected size region was excised for each sample separately. Protein-bound RNA was released via Proteinase K digestion (200 μl NLS elution buffer (20 mM of Tris pH 8.0, 10 mM of EDTA, 2% NLS, 2.5 mM of TCEP), 25 μl H_2_O, 12.5 μl 5 M NaCl, 12.5 μl Proteinase K) for 1 h at 55°C. Following incubation, 250 μl acid-phenol-chloroform were added and samples were subjected to standard phenol-chloroform extraction procedures. Finally, RNA was purified using the ZYMO RNA Clean and Concentrator-5 kit (Zymo Research) and eluted in 11 μl H_2_O.

Next, RNA of the SMI samples was dephosphorylated by incubating with FastAP mix (10 μl H_2_O, 2.5 μl 10x FastAP buffer, 2.5 μl FastAP enzyme, 0.5 μl Murine RNase inhibitor) for 20 min at 37°C. This was followed by another 20 min incubation at 37°C after addition of PNK mix (55 μl H_2_O, 10 μl 10x PNK buffer, 7 μl T4 PNK enzyme, 1 μl Murine RNase inhibitor, 1 μl Turbo DNase, 1 μl 0.1 M DTT). 20 μl MyOne Silane Dynabeads (Thermo Fisher Scientific) were washed twice with RLT buffer, resuspended in 300 μl RLT buffer and added to the sample, together with 10 μl 5 M NaCl and 615 μl ethanol. After 15 min incubation at room temperature, the supernatant was discarded, and beads were washed twice with 75 % ethanol and air-dried. Beads were resuspended in 10 μl H_2_O and 5 μl supernatant were transferred into a new tube after 5 min incubation at room temperature. After addition of 1.5 μl DMSO and 0.5 μl RiL-19, samples were incubated at 65°C for 2 min, before transferring them to an ice bucket. Ligation was performed for 1 h 15 min at 23°C with intermittent shaking after adding ligation mix (8 μl 50% PEG8000, 2 μl 10x RNA ligase buffer (with DTT), 1.5 μl H_2_O, 1.3 μl T4 RNA ligase high concentration, 0.3 μl DMSO, 0.2 μl Murine RNase inhibitor, 0.2 μl 0.1 M ATP). Following ligation, another cleanup was performed starting with 20 μl MyOne Silane Dynabeads that were washed twice in RLT buffer and resuspended in 61.6 μl RLT buffer, before adding them to the sample. Next, 61.6 μl ethanol were added to each sample and samples were mixed. After 15 min incubation at room temperature, the supernatant was discarded, and beads were washed twice with 75 % ethanol and air-dried. Beads were resuspended in 10 μl H_2_O and supernatant was transferred into a new tube after 5 min incubation at room temperature.

For each IP and SMI sample, 10 μl eluted RNA were mixed with 0.5 μl AR17 primer, and incubated for 2 min at 65°C. Samples were then incubated with reverse transcriptase (RT) mix (4 μl H_2_O, 2 μl 10x Affinity Script buffer, 2 μl DTT, 0.6 μl Affinity Script Enzyme, 0.8 μl 100 mM dNTPs, 0.3 μl Murine RNase inhibitor) for 45 min at 55°C. Following reverse transcription, 3.5 μl ExoSAP-IT (Thermo Fisher Scientific) were added and incubated for 15 min at 37°C. Next, 1 μl 0.5 M EDTA and 3 μl 1 M NaOH were added and samples were incubated for 12 min at 70°C. Samples were then mixed with 3 μl 1 M HCl. Next, 10 μl MyOne Silane Dynabeads were washed twice in RLT buffer, resuspended in 93 μl RLT buffer, and added to the sample together with 111.6 μl ethanol. After 5 min, supernatant was discarded, and beads were washed twice with 80 % ethanol before being air-dried. Beads were resuspended in an elution mix (5 μl H_2_O, 0.8 μl Rand103Tr adapter, 1 μl DMSO) and incubated for 5 min. Samples, including beads, were heated for 2 min at 75°C, cooled on ice and ligation mix (9 μl 50% PEG8000, 10x RNA ligase buffer with DTT, 1.5 μl high concentration T4 RNA ligase, 1.1 μl H_2_O, 0.2 μl 0.1 M ATP) was added and incubated overnight, shaking at 22°C. 5 μl fresh Dynabeads MyOne Silane (Thermo Fisher Scientific) were washed twice with RLT buffer, resuspended in 60 μl RLT buffer, and added to the sample together with 60 μl ethanol. After 5 min incubation, beads were washed three times in 75 % ethanol, and air-dried. DNA was eluted in 45 μl H_2_O for 5 min and 43 μl supernatant were subsequently transferred into new PCR tubes. From each sample, 21 μl were mixed with 25 μl 2x Q5 Hot start master mix and 2 μl universal forward PCR primer (25 μM) and 2 μl barcoded reverse PCR primer (25 μM) and amplified with 12-15 PCR cycles. PCR products were mixed with 90 μl AmpureXP bead suspension and incubated for 10 min. Beads were washed twice in 75% ethanol and air-dried. DNA was eluted in 21 μl H_2_O for 5 min. DNA was separated on a 2% agarose gel and the expected library size (175-250 bp) was excised from the gel followed by gel purification using the Zymoclean Gel DNA Recovery kit (Zymo Research). Concentration was determined using Qubit dsDNA Hs reagent and libraries were subsequently sequenced using the Illumina NextSeq platform.

#### Covalent protein capture and sequencing of crosslinked RNA

To capture RNA sequences covalently crosslinked to proteins purified with RAP-MS, we carried out RNA antisense purifications as described above and isolated 5% of the washed beads prior to protein elution. Purified protein-RNA complexes were released from streptavidin beads by RNase H treatment using 7.5 μl RNase H (NEB), 2 μl TURBO DNase (Thermo Fisher Scientific) in 55.5 μl RNase H buffer (100 mM HEPES pH 7.5, 75 mM NaCl, 3 mM MgCl_2_, 0.125% NLS, 0.025% sodium deoxycholate, 2.5 mM TCEP) and incubated for 30 min at 37°C. Following elution of proteins, supernatants were transferred into new tubes and beads were washed once with RNase H buffer to aid the release of digested protein-RNA complexes. Wash fractions were pooled with eluates and stored on ice. The next steps were performed as previously described.^91^ Briefly, we separated 100 μl of NHS-activated magnetic beads (Thermo Fischer Scientific) on a magnet and discarded the supernatant. We then washed the beads with 1 ml 1 mM ice-cold HCl, followed by a quick rinse in 1 ml ice-cold PBS. After removing PBS, we immediately added the stored eluates to the prepared beads. Binding was carried out overnight at 4°C on a rotating wheel. The next day, we quenched NHS beads by adding 1 ml of 0.5 M Tris pH 8.0 and incubating for 1 h at 4°C. We then washed beads four times in 1 ml of modified RLT buffer (RLT buffer supplied by Qiagen with added 10 mM Tris pH 7.5, 1 mM EDTA, 1 mM EGTA, 0.2% NLS, 0.1% Triton-X, and 0.1% IGEPAL CA-630). Next, we washed beads two more times in 1 ml 1x PBS, 5 mM EDTA, 5 mM EGTA, 5 mM DTT, 0.2% Triton-X, 0.2% IGEPAL CA-630, 0.2% sodium deoxycholate and incubated each washing step 5 min at 55°C. These heated washing steps were followed by two additional washes in the same buffer at room temperature. Subsequently, beads were rinsed on the magnet in 1x FastAP buffer (10 mM Tris pH 7.5, 5 mM MgCl_2_, 100 mM KCl, 0.02% Triton X-100). Next, end repair was carried out by resuspending beads in 50 μl FastAP mix (39 μl H_2_O, 5 μl 10x FastAP buffer (Thermo Fisher Scientific), 1 μl Murine RNase Inhibitor (NEB), 5 μl FastAP enzyme (Thermo Fisher Scientific) and incubating 20 min at 37°C. In the meantime, we prepared 150 μl of T4 PNK mix (120 μl H_2_O, 20 μl 10x T4 PNK buffer (NEB), 1 μl Murine RNase Inhibitor (NEB), 7 μl T4 PNK (NEB), 1 μl TURBO DNase (Thermo Fisher Scientific)), which was added to the FastAP reaction and incubated for another 20 min at 37°C. Following end-repair, we washed beads once in modified RLT buffer, and two times in Detergent Wash Buffer (20 mM Tris pH 7.5, 50 mM NaCl, 0.2% Triton-X, 0.2% IGEPAL CA-630, 0.2% sodium deoxycholate). We then rinsed beads on the magnet twice with 1x T4 RNA ligase buffer (50 mM Tris-HCl pH 7.5, 10 mM MgCl_2_), before resuspending the beads in 25 μl RNA ligation mix (9 μl H_2_O, 3 μl 10x T4 RNA ligase buffer (NEB), 0.3 μl 0.1 M ATP, 0.8 μl DMSO, 0.4 μl Murine RNase Inhibitor (NEB), 9 μl PEG 8000, 2.5 μl T4 RNA ligase I High Concentration (NEB). Next, we added 5 μl of 20 nM RiL-19 and incubated samples for 75 min at 23°C. Following 3ʹ-end ligation, we washed the beads once in 1 ml modified RLT Buffer, followed by two washes in Detergent Wash Buffer. Next, we resuspended beads in 250 μl Proteinase K mix containing 200 μl NLS RNA Elution buffer (20 mM Tris pH 8.0, 10 mM EDTA, 2% NLS, 2.5 mM TCEP), 12.5 μl 5 M NaCl, 1 μl 500 mM TCEP, 12.5 μl Proteinase K (NEB), and 24 μl H_2_O and incubated samples for 1.5 h at 55°C. Following Proteinase K digestion, we separated beads on a magnet, transferred the supernatant into a new tube and extracted RNA using Phenol/Chloroform extraction. All subsequent manipulation steps were carried out as described in the eCLIP library preparation protocol described above,^41^ starting with the reverse transcription of recovered RNA fragments.

#### SLAM-seq

##### RNA labeling, extraction, and alkylation

A549^ACE2^ SND1 knockout cells, control cells, or parental A549^ACE2^ wild-type cells were infected with SARS-CoV-2 at MOI 3 PFU/cell. For each time point outlined in Figure 4B, 4-Thiouridine (Biosynth Carobsynth, NT06186) was added to the cell culture medium to a final concentration of 200 μM and cells were returned to the incubator for 2 h. Cells were harvested in Trizol and RNA was extracted using the Direct-zol RNA microprep kit (Zymo Research) including DNase digest. From each sample, 2 μg of RNA were subjected to alkylation by incubating with 10 mM IAA (Thermo Fisher Scientific, A39271) in PBS with 50% DMSO at 50°C for 15 min as previously described.^44^ After adding an equal volume of H_2_O to the sample, the RNA was purified using the RNA Clean and Concentrator-5 kit (Zymo Research).

##### Library preparation

Approximately 800 ng of alkylated RNA was depleted of ribosomal RNA using the NEBNext rRNA Depletion Kit v2 (NEB, E7400X) according to the manufacturer’s instructions. RNA was heat fragmented at 91°C for 3 min in 10 mM Tris-HCl pH 8.0, 5 mM MgCl2, 100 mM KCl, 0.02% Triton X-100 and 0.1 mg/mL BSA. RNA was dephosphorylated using 1.75 U FastAP Thermosensitive Alkaline Phosphatase (Thermo Fisher Scientific, EF0651) for 10 min at 37°C, and end-repaired using 15 U T4 PNK (NEB, M0201L) for 20 min at 37°C. Both reactions were performed in the presence of 10 U murine RNase inhibitor (NEB, M0314L). RNA was cleaned up using Dynabeads MyOne Silane (Thermo Fisher Scientific, 37002D). For this, 10 μl beads per sample were washed twice with RLT buffer (Qiagen, 79216) and beads were added to the RNA in 3x sample volumes RLT buffer. To bind the RNA to the beads, 4.5x volumes isopropanol were added and samples were incubated 5 min at room temperature. Beads were washed twice with 70% EtOH and RNA was eluted in 8 μl H_2_O. Poly-A-tailing of the fragmented and end-repaired RNA was performed using 0.5 U Poly-A-polymerase (Takara, 2180A) in Smartscribe first stand buffer (Takara, 639538) supplemented with 0.1 mM ATP (NEB, M0494L) and 5 U SUPERase-In RNase Inhibitor (Thermo Fisher Scientific, AM2696). RNA was mixed with 100 U SMARTScribe Reverse Transcriptase (Takara, 639538), 0.5 μM RT primer, 1 mM deoxynucleotide mix (Agilent Technologies, 200415) and 0.5 mM DTT, and incubated at 42°C for 15 min. Next, the template-switching oligo was added to a final concentration of 0.5 μM, and the reaction was incubated for another 90 min at 42°C, followed by a 10 min incubation at 70°C. The cDNA was PCR amplified using Q5 Hot Start High-Fidelity 2X Master Mix (NEB, M0494L), 1 μM universal forward PCR primer and 1 μM barcoded reverse PCR primer. The cDNA library was purified from the reaction using 0.8 volumes AMPure XP beads (Beckmann Coulter, A63881), followed by gel purification of the fragments migrating between 175 bp and 350 bp in size with the Zymoclean DNA Gel Recovery Kit (Zymo Research, D4007). Concentration was determined using Qubit Hs reagent and libraries were subsequently sequenced using the Illumina NextSeq platform.

#### Co-immunoprecipitation and mass spectrometry

##### Infection, cell lysis, and immunoprecipitation

For each Co-IP, 20 million A549^ACE2^ cells were infected with SARS-CoV-2 at MOI 5 PFU/cell or mock infected. At 24 h post-infection, cells were rinsed with cold PBS, collected by scraping and freshly lysed in 500 μl 2x Co-IP lysis buffer (100 mM Tris-HCl pH 7.4, 300 mM NaCl, 2% IGEPAL CA-630, 1% Sodium Deoxycholate, 0.5 mM TCEP, EDTA-free Protease Inhibitor Cocktail (Thermo Fisher Scientific)). After 30 min incubation at room temperature, 500 μl H_2_O was added and lysates were cleared by centrifugation at 14,000 g for 15 min at 4°C to remove cell debris. The resulting lysates were pre-cleared by incubating them with uncoupled Protein G beads (Thermo Fisher Scientific, 10004D). We determined the total protein concentration in pre-cleared lysates by BCA assay in triplicates and normalized all Co-IP samples to contain exactly 2 mg of total protein. Samples for total proteome measurements were put aside. For every mg of total protein, 5 μg antibody were coupled to 30 μl Protein G beads for 1 h at room temperature (anti-SND1-antibody: Bethyl, A302-883A; normal rabbit IgG: Cell Signaling, 2729). The antibody-coupled beads were added to the pre-cleared lysate for overnight incubation at 4°C on a rotating wheel. The next day, supernatant was removed and beads were washed twice in 50 mM Tris-HCl pH 7.5, 150 mM NaCl and 0.05% IGEPAL CA-630, followed by two washes in 50 mM Tris-HCl pH 7.5 and 150 mM NaCl. After the last wash, beads were eluted with 1x LDS sample buffer (Thermo Fisher Scientific, B0007) prior to mass spectrometry sample preparation.

##### Protein digestion and TMT labeling

Disulfide bonds were reduced with 10 mM DTT at 56°C for 20 min in 50 mM HEPES pH 8.5 and alkylated with 20 mM 2-chloroacetamide at 24°C for 20 min in 50 mM HEPES pH 8.5. Samples were cleaned up according to the SP3 protocol.^92^^,^^93^ Sequencing grade Trypsin (Promega) was added in an enzyme to protein ratio of 1:50 and incubated overnight at 37°C. Peptides were labelled with TMT10-plex^94^ (Co-IP samples) or TMT6-plex^95^ (input lysates) Isobaric Label Reagent (ThermoFisher) according to the manufacturer’s instructions. In short, 0.8 mg reagent was dissolved in 42 μl 100% acetonitrile and 8 μl of stock was added and incubated for 1 h room temperature. The reaction was quenched with 5% hydroxylamine for 15 min. Samples were combined and desalted on an OASIS® HLB μElution Plate (Waters). Offline high pH reverse phase fractionation was carried out on an Agilent 1200 Infinity high-performance liquid chromatography system, equipped with a Gemini C18 column (3 μm, 110 Å, 100 x 1.0 mm, Phenomenex).^96^ Thirty-two fractions were collected and pooled into 6 fractions (Co-IP samples) or 12 fractions (input lysates), dried under vacuum centrifugation, reconstituted in 10 μl 1% formic acid, 4% acetonitrile for LC-MS analysis.

##### LC-MS/MS analysis

An UltiMate 3000 RSLC nano LC system (Dionex) equipped with a trapping cartridge (C18 PepMap 100, 5μm, 300 μm i.d. x 5 mm, 100 Å) and an analytical column (nanoEase M/Z HSS T3 column 75 μm x 250 mm C18, 1.8 μm, 100 Å, Waters) was coupled directly to an Orbitrap Fusion Lumos Tribrid Mass Spectrometer (Thermo) using the Nanospray Flex ion source in positive ion mode. The peptides are concentrated on the pre-column with a constant flow of 0.05% trifluoroacetic acid in water at 30 μl/min for 6 minutes. The analytical column is operated with a constant flow of 0.3 μl/min and a binary solvent system (solvent A: 0.1% formic acid in water, 3% DMSO; solvent B: 0.1% formic acid in acetonitrile, 3% DMSO) is used for elution of the peptides over a gradient from 2% to 8% in 6 min, from 8% to 26% in 42 min (lysates, for Co-IP in 72 min), from 26% to 38% in 4 min, followed by an increase of B from 38 to 80% in 4min and a re-equilibration back to 2% B for 4 min. The peptides were introduced into the Fusion Lumos via a fused silica emitter 360 μm OD x 20 μm ID; 10 μm tip (CoAnn Technologies) and an applied spray voltage of 2.4 kV. The capillary temperature was set at 275°C. Full mass scan (MS1) was acquired with mass range 375-1500 m/z in profile mode in the orbitrap with resolution of 60000 (lysates, for Co-IP 120,000). The filling time was set at maximum of 50 ms. Data dependent acquisition (DDA) was performed with the resolution of the Orbitrap set to 15000 (lysates, for Co-IP 30000), with a fill time of 54 ms (lysates, for Co-IP 94 ms) and a limitation of 1x10^5^ ions. A normalized collision energy of 36 was applied. Fixed first mass m/z 110. MS2 data were acquired in profile mode.

#### Hybridization-chain-reaction RNA-FISH staining

Approximately 3x10^4^ A549^ACE2^ cells were seeded per well of a 12-well chamber slide (IBIDI, 81201) and infected the next day with SARS-CoV-2 at MOI 3 PFU/cell. At 8 hpi, the cells were washed 1x with PBS and fixed in 4% methanol-free formaldehyde (Thermo Fisher Scientific, 28908) for 20 min at room temperature. We performed HCR staining for SARS-CoV-2 N RNA (negative-sense) using the HCR IHC + HCR RNA-FISH Kit (Molecular Instruments). The protocol was performed according to the manufacturer’s instructions with the following modifications: All volumes were adapted to 100-150 μl per well of the 12-well chamber and probes were used at a final concentration of 4 nM. For detection of negative-sense viral RNA, we performed a denaturation step before adding the split initiator probes: The cells were incubated with pre-heated 90% DMSO at 70°C for 1 h, cooled on ice and washed once with ice-cold PBS, followed by two washes with PBS at room temperature.^64^ For combining immunostaining with HCR-based detection, we used anti-J2 antibody (Jena Bioscience, RNT-SCI-10010200, 1:500) or anti-SND1 antibody (Proteintech, 60265-1- Ig, 1:500). Incubation with the primary antibody was performed for 1 h at room temperature. The amplification step was performed for 2 h at room temperature. Nuclei were stained with DAPI (Thermo Fisher Scientific, D1306, 1 μg/ml) and slides were mounted using 100 μl Fluoromount-G mounting medium (Thermo Fisher Scientific, 00495802).

#### Proximity ligation assay

PLA experiments were performed using the Duolink *in situ* Proximity Ligation Assay (Sigma Aldrich, DUO92101). A549^ACE2^ cells were seeded, infected, and fixed as described for HCR RNA-FISH staining. Chambers were washed twice with PBS and subsequently permeabilized with 0.5% Triton-X-100 for 5 min at room temperature. After three washes with PBS, the cells were blocked with Duolink Blocking Solution for 60 min at 37°C. Blocking solution was replaced with Antibody Diluent, containing primary antibodies and slides were incubated overnight at 4°C. Chambers were washed three times with Wash Buffer A for 5 min and incubated with PLA probes in Antibody Diluent for 1 h at 37°C. Next, chambers were washed three times with Wash Buffer A for 5 min and ligation mix was added, followed by incubation for 30 min at 37°C. After three washes, with Wash Buffer A for 5 min, samples were incubated with Amplification Buffer for 100 min at 37°C and washed three times with Wash Buffer B for 10 min, followed by a wash with 0.01x Wash Buffer B for 1 min. Slides were mounted using Duolink *In Situ* Mounting Medium with DAPI. The following antibodies were used at indicated dilutions: anti-SND1 (Proteintech, 60265-1-Ig, 1:500), anti-NSP9 (GeneTex, GTX135732, 1:2000).

#### Immunofluorescence staining of infected cells

Cells were seeded, infected, and fixed as described for HCR RNA-FISH staining. If cells were not processed immediately, they were stored in PBS at 4°C. Chambers were washed twice with PBS and subsequently permeabilized by incubating with 0.5% Triton-X-100 for 5 min at room temperature. After three washes with PBS, the cells were blocked with PBS containing 2% FBS for 30 min at room temperature. Next, cells were incubated with 100 μl PBS, containing 2% FBS and all primary antibodies for 1 h at room temperature. Afterwards, the cells were washed three times 2 min with PBS and then incubated with 100 μl PBS, containing 2% FBS and secondary antibodies for 1 h at room temperature in the dark. After washing three times with PBS, the cells were stained with DAPI (Thermo Fisher Scientific, D1306, 1 ug/ml) for 2 min and subsequently washed another three times with PBS. The cells were quickly washed with H_2_O and then mounted, using 100 μl Fluoromount-G mounting medium (Thermo Fisher Scientific, 00495802). After drying at room temperature, the slides were stored at 4°C. The following antibodies were used: anti-dsRNA J2 (Jena Bioscience, RNT-SCI-10010200, 1:500), anti-SND1 (Proteintech, 10760-1-AP, 1:1000), anti-SARS-CoV-2 NSP9 (GeneTex; GTX135732; 1:2000), anti-SARS-CoV-2 NSP3 (GeneTex; GTX135614; 1:250).

#### Imaging

Fluorescent microscopy images of PLA-stained samples were acquired using the Leica Thunder Imager system and Leica LAS X software. Confocal microscopy of immunofluorescence and HCR-stained samples was performed using the Leica TCS SP5 system.

#### High pressure freezing, freeze substitution, and ultramicrotomy

Sapphire discs with 3.0 mm diameter and 50 μm thickness (Wohlwend GmbH) were cleaned with 100% ethanol and coated with a 15 nm carbon layer using a sputter coater (EM ACE600, Leica). An “F” letter was scratched on the carbon side to distinguish between the two sides of the disc and baked overnight at 120°C. Prior to use, the carbon-coated discs were pre-soaked in DMEM in a 35 mm dish with the “F” side facing up. The DMEM was removed and 4x10^5^ A549^ACE2^ CTRL or A549^ACE2^ SND1 KO cells per dish were seeded on the sapphire discs. Cells were infected the next day with SARS-CoV-2 at MOI 10 PFU/cell. At 8 hpi, infected cells were washed with PBS and fixed in 4% formaldehyde and 0.1% glutaraldehyde in PHEM buffer (60 mM PIPES, 25 mM HEPES, 10 mM EGTA, 2 mM MgCl_2_). Sapphire discs with infected cells were assembled with 1-hexadecene coated specimen carrier Type A and B (Wohlwend GmbH) with cells facing the 100 μm deep cavity of carrier Type A. Cells were vitrified by high pressure freezing at minimum 2,200 bar maintained for 370 ms with a cooling rate of 20,000K/s using a Leica EM ICE. Sapphire discs with vitrified cells were transferred from liquid nitrogen to a freeze-substitution (FS) solution (0.1% uranyl acetate in anhydrous acetone) cooled to -90°C and processed in automated FS system (EM AFS2, Leica). After washing with acetone, samples were infiltrated with Lowicryl HM20 and polymerized using UV light. The FS protocol was performed as previously described.^97^ Lowicryl-embedded samples were sectioned using diamond knife (DiATOME) at an ultramicrotome (UC7, Leica). Sections with 200 nm nominal thickness were placed on 2 × 1 mm copper slot grids (Gilder) coated with pioloform support film.

#### Electron tomography of resin sections

Grids were imaged with a Talos L120C TEM operated at 120 keV and equipped with a LaB_6_ filament and a Ceta-M camera with a 4k × 4k CMOS (Thermo Fisher Scientific). Unidirectional tilt series (TS) were recorded at a magnification of 45,000 × (corresponding to pixel size at the specimen level of 3.28 Å) from +60° to -60° with 2° increments using target counts 4000 and target defocus -0.7 μm. Mapping and data acquisition was performed in SerialEM.^98^

#### Tomogram reconstruction and analysis

TS were aligned using patch tracking and reconstructed with the IMOD software package.^99^ The aligned TS was filtered using a Fourier low pass filter with high-frequency cutoff set to 0.25 and falloff 0.035. The final reconstruction was performed by weighted back-projection with a Simultaneous Iterative Reconstruction Technique (SIRT)-like filter equivalent to 5 iterations and with Fourier low pass filter with high-frequency cutoff set to 0.25 and falloff 0.035. DMV diameter measurements were performed in 3D on reconstructed tomograms in IMOD. We calculated the ellipse area using diameter measurements along the long and short axis.

#### Microscale thermophoresis

Site-specific cysteine labeling of SND1 was performed using the RED-MALEIMIDE 2nd Generation kit (Nanotemper) according to the manufacturer‘s instructions. For each binding experiment, recombinant SND1 (Origene, TP300059) was diluted to 10 nM in Buffer A (50 mM Tris Cl pH 7.4, 150 mM NaCl, 2 mM DTT, 0.05% Tween 20, 5% glycerol). A series of 16 tubes with recombinant NSP9 (Abcam, ab289608) dilutions were prepared in Buffer A, producing NSP9 ligand concentrations ranging from 254 pM to 8.33 μM. For measurements, each ligand dilution was mixed with one volume of labeled SND1. The reaction was mixed by pipetting, incubated for 15 min at room temperature, followed by centrifugation at 17,000 × *g* for 5 min in 4^0^C. Capillary forces were used to load the samples into Monolith NT.115 Premium Capillaries (NanoTemper Technologies). Measurements were performed using a Monolith Pico instrument (NanoTemper Technologies) at an ambient temperature of 25 °C. Instrument parameters were adjusted to 5% LED power, medium MST power, and MST on-time of 10 s. An initial fluorescence scan was performed across the capillaries to determine the sample quality and afterward, 16 subsequent thermophoresis measurements were performed. Data of three independently pipetted measurements were analyzed for the ΔFnorm values determined by the MO.Affinity Analysis software (NanoTemper Technologies). Graphs were plotted and binding affinities (linear regression model – one site specific model) were calculated using GraphPad Prism 9.2.0 software.

### Quantification and statistical analysis

#### RNA antisense purification

##### Data analysis

RNA-seq libraries from purified genomic (gRNA) and subgenomic mRNA (sgmRNA) samples were sequenced on an Illumina NextSeq instrument in paired-end mode with a read length of 2 x 40 nucleotides. Each experiment was performed on two independent biological replicates. Paired-end sequencing reads were adapter- and quality trimmed using cutadapt (v1.18). Reads with a total length less than 18 nt were discarded. The trimmed reads were aligned to the genome sequences of human (hg38, Ensembl release 106) and SARS-CoV-2 (NC_045512.2, GenBank: MN908947.3) using STAR (v2.7.10a)^87^ with the parameters –outFilterScoreMinOverLread 0 --outFilterMatchNminOverLread 0 --outFilterMatchNmin 0 --outFilterType Normal --alignSoftClipAtReferenceEnds No --alignSJoverhangMin 8 --alignSJDBoverhangMin 1 --outFilterMismatchNoverLmax 0.04 --scoreDelOpen -1 --alignIntronMin 20 --alignIntronMax 3000 --alignMatesGapMax 3000 --alignEndsType EndToEnd.

Testing the enrichment of crosslinked RNA fragments for gRNA and sgmRNA purification samples, the SARS-CoV-2 genome was divided into two genomic intervals, one characteristic for genomic RNAs (nt: 1 – 21562), and the other characteristic for subgenomic RNAs (nt: 21562 - 29903). Reads aligning to the two intervals were counted by featureCounts^100^ in a strand unspecific manner. Library normalization and calculation of significant differential enrichment of RNA fragments in the two intervals was performed applying the DESeq2^101^ approach using the Wald test statistic for calculating p values.

Calculating the enrichment of RNA fragments derived from the positive or negative strand of the sgmRNA and gRNA purification experiments, all paired-end reads were counted with respect to their strand orientation. Based on the DESeq2 approach the enrichments of RNA fragments originating from the positive strand and RNA fragments originating from the negative strand were calculated between the two purification experiments, using the Wald test statistic for calculating p values.

#### Mass spectrometry data analysis for RAP-MS

##### Quantification and identification of peptides and proteins

MS/MS spectra were searched on the Spectrum Mill MS Proteomics Workbench against a RefSeq-based sequence database containing 41,457 proteins mapped to the human reference genome (hg38) obtained via the UCSC Table Browser (https://genome.ucsc.edu/cgi-bin/hgTables) on June 29, 2018,with the addition of 13 proteins encoded in the human mitochondrial genome, 264 common laboratory contaminant proteins, 553 human non-canonical small open reading frames, 28 SARS-CoV-2 proteins obtained from RefSeq derived from the original Wuhan-Hu-1 China isolate NC_045512.2,^102^ and 23 novel unannotated SARS-CoV-2 ORFs whose translation is supported by ribosome profiling,^103^ yielding a total of 42,337 proteins. Amongst the 28 annotated SARS-CoV-2 proteins we opted to omit the full-length ORF1ab, to simplify peptide-to-protein assignment, and instead represented ORF1a and ORF1ab as the mature 16 individual non-structural proteins that result from proteolytic processing of the 1a and 1ab polyprotein. Finally, we added to the database the SwissProt entry for ORF9b. We further added the D614G variant of the SARS-CoV-2 Spike protein that is commonly observed in European and American virus isolates. Spectrum Mill search parameters included: instrument setting of ESI-QEXACTIVE-HCD-v4-35-20, parent and fragment mass tolerance of 20 ppm, trypsin allow P enzyme setting and up to 4 missed cleavages. Carbamidomethylation and TMT labeling at Lysine (with and without labeling at N terminus) were set as fixed modifications, while variable modifications included acetylation of protein N-termini, oxidized methionine, deamidation of asparagine, and pyro-glutamic acid at peptide N-terminal glutamine. Peptide spectrum match score thresholding was optimized to achieve a target-decoy false discovery rate (FDR) of 1.2% and 0.6% for validation of charges 2-4 and charge 5 spectra, respectively. Peptide level auto-validation was followed by protein polishing with FDR of 0% at protein level and a minimum score of 13.

##### Statistical data analysis

The Spectrum Mill generated proteome level export were filtered for human proteins identified by two or more distinct peptides, SARS-CoV-2 proteins and unannotated virus ORFs were used for further statistical analyses. Keratins were excluded. Protein quantification was achieved by computing the ratio of TMT reporter ion for each of all viral RNA, gRNA and sgmRNA samples relative to RMRP. Additionally, the ratio of sgmRNA relative to gRNA was also computed and used to compare these samples directly. TMT10 reporter ion intensities were corrected for isotopic impurities in the Spectrum Mill protein/peptide summary module using the afRICA correction method which implements determinant calculations according to Cramer's Rule and correction factors obtained from the reagent manufacturer’s certificate of analysis (https://www.thermofisher.com/order/catalog/product/90406) for lot number TE270748 or UA280170. Moderated one-sample t-test was applied to all the sample groups after median-MAD or median normalization for A549^ACE2^ and Huh-7 experiments, respectively. Benjamini-Hochberg corrected *P value* threshold of 0.05 was used to assess significantly regulated proteins in each of the datasets.

#### Mass spectrometry data analysis for CoIP and total proteome

##### Quantification and identification of peptides and proteins

IsobarQuant^104^ and Mascot (v2.2.07) were used to process the acquired data, which was searched against a combined Uniprot proteome database of *Homo sapiens* proteome database (UP000005640) and *SARS-CoV-2* (UP000464024) containing common contaminants and reversed sequences. Carbamidomethyl on cystein and TMT6 on lysine (lysates, for Co-IP: TMT 10) were set as fixed modifications. Acetyl (Protein N-term), Oxidation (M) and TMT6 on N-termini (lysates, for Co-IP TMT10) were set as variable modifications. For the full scan (MS1) a mass error tolerance of 10 ppm and for MS/MS (MS2) spectra of 0.02 Da was allowed. As protease Trypsin was chosen with maximum of two missed cleavages. Further parameters were: a minimum peptide length of seven amino acids: false discovery rate on peptide and protein level: 0.01.

##### Statistical data analysis

The raw output files of IsobarQuant (protein.txt – files) were processed using the R programming language. Only proteins that were quantified with at least two unique peptides were considered for the analysis. Raw TMT reporter ion signals (signal_sum columns) were first cleaned for batch effects using limma^105^ and further normalized using vsn (variance stabilization normalization^106^). During the normalization of the IP experiment, different normalization coefficients were estimated for the IgG and SND1 conditions in order to maintain the abundance difference. Proteins were tested for differential expression using the limma package. The replicate information was added as a factor in the design matrix given as an argument to the ‘lmFit’ function of limma.

#### SLAM-seq data analysis

SLAM-seq was performed in duplicates to identify newly synthesized RNA and total RNA using GRAND-SLAM.^45^ Sequencing adapters were trimmed using Cutadapt (version 3.4). Next, bowtie2 (version 2.3.0)^107^ with default parameters was used to discard reads mapping to rRNA (Genbank identifier U13369.1) and to verify the absence of mycoplasma contamination. STAR version 2.5.3a^87^ was used to map all remaining reads with length at least 18 nt against a combined index of the human genome (hg38/Ensembl version 90) and the SARS-CoV-2 genome (NC_045512) using STAR (version 2.5.3a) with the parameters –outFilterMismatchNmax 20 –outFilterScoreMinOverLread 0.4 –outFilterMatchNminOverLread 0.4 –alignEndsType Extend5pOfReads12 –outSAMattributes nM MD NH. We used GRAND-SLAM (version 2.0.6) to estimate the new-to-total RNA ratios. New RNA was computed by multiplying total RNA with the maximum a posteriori estimate of the new-to-total RNA ratio. Log2 fold changes were estimated using PsiLFC.^108^ Normalization factors were computed from total RNA such that the median log_2_ fold change was 0 and applied to both total and new RNA.

#### cRIP and eCLIP analysis

cRIP and eCLIP libraries were sequenced on an Illumina NextSeq instrument in paired-end mode with a read length of 2 x 40 nucleotides. Paired-end sequencing reads from cRIP or eCLIP experiments, were adapter- and quality trimmed using cutadapt (v1.18).^109^ Reads with a total length less than 18 nt were discarded. A custom java program was applied that simultaneously identified and clipped the remaining unique molecular identifier (UMI) associated with each read. These trimmed reads were then aligned to the human (hg38, Ensembl release 106) and the SARS-CoV-2 reference genome (NC_045512.2, GenBank: MN908947.3) using STAR (v2.7.10a)^87^ with the parameters –outFilterScoreMinOverLread 0 –outFilterMatchNminOverLread 0 –outFilterMatchNmin 0 –outFilterType Normal –alignSoftClipAtReferenceEnds No --alignSJoverhangMin 8 –alignSJDBoverhangMin 1 –outFilterMismatchNoverLmax 0.04 --scoreDelOpen -1 –alignIntronMin 20 --alignIntronMax 3000 –alignMatesGapMax 3000 –alignEndsType EndToEnd. Next, we removed PCR duplicates using the UMI-aware deduplication functionality in Picard’s MarkDuplicates. The resulting alignment reads were separated based on their strand orientation. Regions with enriched protein binding were identified for each strand with MACS2^85^ by modeling the fold change in IP samples over a paired SMI control using the parameters -g 29903 -s 31 --keep-dup all --nomodel --d-min 25 --call-summits --scale-to small --shift 25 --nolambda --extsize 5 --max-gap 20 --min-length 5. The identified MACS2 peaks were additionally filtered by calculating the enrichment of strand specific reads within each peak over all remaining strand specific mapped reads between IP and size matched input. A statistically significant enrichment relative to SMI control was calculated by a one-sided Fisher’s exact test. The resulting *P value*s were corrected with the Benjamini-Yekutieli^110^ procedure and only peaks with an adjusted P value < 0.05 were considered for further downstream analysis.

The enrichment analysis of positive and negative-strand viral RNAs was performed on the number of reads mapped in positive and negative sense orientation for each strand-specific peak as follows. A Poisson distributed random variable *X*_*peak*_ was defined for each peak, measuring the number of reads mapping within this interval. Further, for each peak, the variables *x*_*strand*_ and λ_*opposite*_ were defined. The variable *x*_*strand*_ denotes the number of reads mapping with the same strand orientation as the observed peak interval (either positive or negative). The variable λ_*opposite*_ denotes the number of reads mapping within the same peak interval but with opposite strand orientation. Suppose *x*_*strand*_ represents the number of reads mapping in positive-sense orientation within a positive-sense peak interval. In that case, λ_*opposite*_ represents the number of reads mapping in negative-sense orientation within this interval. The enrichment analysis was based on the Poisson distributed random variable *X*_*peak*_ *∼ Poisson(*λ_*opposite*_*)* by testing the null hypothesis *H*_*0*_*: x*_*strand*_ *=* λ_*opposite*_ (alternative *H*_*1*_*: x*_*strand*_ *>* λ_*opposite*_). The corresponding *P value* was calculated as the probability *P(X*_*peak*_ > *x*_*strand*_ | λ_*opposite*_*)*. To account for multiple testing, the resulting *P value*s were corrected using the Benjamini-Yekutieli procedure. The null hypothesis was rejected if the corrected *P value* was below a significance level of 0.05 and a significant enrichment could be assumed.

To identify regions with a statistically significant change in NSP9 binding between SND1 knockout and control cell lines, overlapping peak intervals were calculated within each time point by using *bedtools intersect*.^84^ For each intersected interval, a two-sided Fisher’s exact test was performed between the two conditions comparing the number of mapped reads within the peak interval to the number of reads mapped in the remainder of the viral genome. The resulting *P value*s were corrected using the Benjamini-Yekutieli procedure. Adjusted *P value*s below a threshold of 0.05 were considered statistically significant.

Crosslinking sites were defined as the first nucleotide in 5ʹ-direction at the 5ʹ-end of a R2 read that overlaps a significantly enriched peak. The coverage of each crosslinking-site in IP and SMI was calculated as the number of R2 reads sharing the same 5ʹ-end. Statistically enriched crosslinking sites in IP were calculated by using the Fisher’s exact test in the same manner as described for an enriched peak in IP over SMI. The resulting *P value*s were corrected using the Benjamini-Yekutieli procedure. Adjusted *P value*s below a threshold of 0.05 were considered statistically significant.

The overall eCLIP and cRIP signals were visualized by calculating the relative information content of IP over SMI.^111^^,^^112^^,^^113^ The relative information content was defined as *p*_*i*_ x *log*_*2*_*(p*_*i*_/*q*_*i*_*)*, where *i* denotes a certain genomic position, *p*_*i*_ represents the fraction of total aligned reads in IP and *q*_*i*_ represents the fraction of total aligned reads in SMI. Visualizations of the region were rendered from the PCR-deduplicated mapping files using the Integrative Genome Visualization (IGV) Browser.

#### Image analysis

To quantify dsRNA foci detected by J2 antibody staining, z-stacks obtained by confocal microscopy were processed using big-FISH (v 0.6.1 dev) for segmentation and spot detection. First, images were converted to NumPy arrays for segmentation. To segment individual cells, the DAPI channel was used to obtain a binary mask (threshold=20), followed by binary artefacts removal (small_object_size=500, smoothness=10). After the nuclei mask was built, we used the watershed algorithm to identify the cell boundary (threshold=10, alpha=1). Masks that inaccurately detected more than one infected cell were manually excluded. Once single-cell information was obtained, we proceeded with RNA detection. RNA detection includes two steps, signal-to-noise enhancement and spot detection. Images were initially filtered using a Laplacian of Gaussian (LoG) filter (sigma=2.5), followed by local maximum detection. We applied the same threshold to each set of images that were acquired in the same session. Spot counts per cell were extracted and cells with more than 5 detected RNAs were kept for further analysis.

#### Quantification of co-localization

The co-localization analysis was conducted using JACoP (Just Another Co-localization Plugin) in FiJi (ImageJ 2.9.0/1.53t, Java 1.8.0_322). Raw images were imported into FiJi, and Manders’ co-localization coefficient analysis was performed with JACoP, using threshold settings based on uninfected cells as the control.

#### Protein-protein interaction network

To establish protein-protein interactions for the proteins identified in the consensus SARS-CoV-2 RNA interactome in Huh-7 and A549^ACE2^ cells, we utilized STRING v11.^13^ For all network and interaction inferences, we use the “combined score” from STRING, which utilizes both physical and functional interactions. Edges between interacting proteins were included for those above a combined interaction score of 600.

#### Gene ontology enrichment analysis

We performed hypergeometric gene ontology (GO) enrichment analyses for the various SARS-CoV-2 RNA interactomes and protein subgroups specified in the main text using the Database for Annotation, Visualization and Integrated Discovery (DAVID) tool (https://david.ncifcrf.gov/tools.jsp) and applying default settings.^114^^,^^115^

## Data Availability

• Next-generation sequencing data have been deposited at GEO. CoIP MS data have been deposited to the ProteomeXchange Consortium via the PRIDE partner repository. RAP-MS data have been deposited to the public proteomics repository MassIVE. All deposited data are publicly available as of the date of publication. Accession numbers and dataset identifiers are listed in the key resources table. Original western blot images and microscopy data are available from the lead contact upon request. • All original code has been deposited at Github and Zenodo and is publicly available as of the date of publication. DOIs are listed in the key resources table. • Any additional information required to reanalyze the data reported in this paper is available from the lead contact upon request.

## Supplemental information

Table S1. Quantitative mass spectrometry data for subgenome-resolved SARS-CoV-2 RNA interactomes, related to Figure 1

Table S2. Analysis of subgenome-resolved SARS-CoV-2 RNA interactomes, related to Figure 1

Table S3. Analysis of strand preference for SND1 (eCLIP) and NSP9 (cRIP) peaks on viral RNA, related to Figures 3 and 6

Table S4. SND1 protein-protein interactome in SARS-CoV-2 infected and uninfected cells, related to Figure 5

Table S5. Covalent NSP9 binding sites on SARS-CoV-2 RNA at 8 and 12 hpi, related to Figure 6

Table S6. Differential NSP9 peaks and covalent linkage sites in SARS-CoV-2 RNA in SND1 knockout vs. control cells, related to Figure 6
